# Targeted Delivery of Indole‐3‐Pyruvic Acid Suppresses Macrophage Ferroptosis to Enhance CD8^+^ T Cell‐Mediated Immunotherapy Response in Bladder Cancer

**DOI:** 10.1002/advs.76319

**Published:** 2026-07-02

**Authors:** Jianwen Lao, Xinhao Yuan, Shuai Liang, Kai Deng, Daqin Wu, Longhao Xu, Kun Zheng, Yi Lin, Peicong Cai, Haoran Zheng, Junyu Chen, Mingli Luo, Yan Chen, Xiong Chen, Chunhui Wang, Wenlong Zhong

**Affiliations:** ^1^ Department of Urology Sun Yat‐sen Memorial Hospital Sun Yat‐sen University Guangzhou P. R. China; ^2^ Guangdong Provincial Key Laboratory of Malignant Tumor Epigenetics and Gene Regulation Guangdong‐Hong Kong Joint Laboratory for RNA Medicine Sun Yat‐sen Memorial Hospital Sun Yat‐sen University Guangzhou P. R. China; ^3^ Guangdong Provincial Clinical Research Center For Urological Diseases Guangzhou P. R. China; ^4^ Department of Urology Yan'an Hospital Kunming Medical University Kunming P. R. China

**Keywords:** CD8^+^ T cells, ferroptosis, immune checkpoint blockade, indole‐3‐pyruvic acid, macrophage

## Abstract

Intratumoral microbiota‐related metabolites are emerging regulators of tumor immunity, yet their therapeutic potential remains largely unexplored. Here, indole‐3‐pyruvic acid (I3P), a Lactobacillus‐associated tryptophan metabolite, is identified as a molecule associated with immunotherapy response in bladder cancer. Mechanistically, I3P suppresses macrophages ferroptosis and sustains CD8^+^ T cell activity through an AHR–NF‐κB–SLC7A11 signaling axis that maintains macrophages redox homeostasis. Disruption of AHR or NF‐κB signaling abolishes these effects. Notably, liposomal delivery of I3P facilitates efficient targeting of tumor‐associated macrophages and enhances immunotherapy response without apparent toxicity. Together, these findings identify I3P as an immunoregulatory metabolite that potentiates anti‐tumor immunity and support nanoparticle‐mediated delivery as a promising strategy for immunotherapy sensitization in bladder cancer.

## Graphical abstract

A microbiota‐derived metabolite, indole‐3‐pyruvic acid, suppresses macrophage ferroptosis through the AHR–NF‐κB–SLC7A11 axis. This preserves CD8^+^ T cell function in bladder cancer. Macrophage‐targeted nanoparticles enhance indole‐3‐pyruvic acid delivery and overcome resistance to PD‐1 blockade. This study reveals a metabolic strategy to sensitize tumors to immunotherapy.

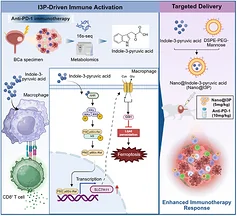

## 1 Introduction

Immune checkpoint blockade (ICB) targeting PD‐1 and PD‐L1 has reshaped the treatment landscape of multiple cancers and produced durable clinical benefit in a subset of patients [1, 2]. However, therapeutic responses remain highly heterogeneous, and only a limited proportion of patients achieve long‐term benefit. Bladder cancer (BCa) is among the tumor types most responsive to immunotherapy, with encouraging activity of PD‐1 blockade reported in both neoadjuvant and adjuvant settings [3, 4]. Nevertheless, substantial inter‐patient variability remains, underscoring the need to better define the mechanisms that govern immunotherapy responsiveness in BCa [5, 6].

Recent microbiome studies have revealed that host‐associated microbial communities play important roles in tumor progression and therapeutic response, partly through modulation of the tumor immune microenvironment (TIME) [7, 8, 9]. In BCa, distinct microbial signatures have been identified in tumor tissues and urine, suggesting that the local microbial ecosystem may actively participate in immune regulation [10, 11]. Among the mechanisms linking microbiota to anti‐tumor immunity, microbe‐derived metabolites have emerged as key functional mediators [12, 13, 14]. Given their abundance and remarkable plasticity within the TIME, tumor‐associated macrophages are well positioned to act as key responders to these microbial cues [15, 16, 17, 18]. Short‐chain fatty acids from microbiota such as butyrate are known to modulate the function and phenotype of TAMs [19], limiting inflammatory cytokine production, skewing macrophage polarization, and regulating cell death programs. This raises the possibility that metabolite‐driven modulation of macrophage death may represent an important interface between microbiota and tumor immunity.

Immune‐cell death is increasingly linked to tumor immunity and ICB response [20]. In particular, ferroptosis has emerged as an important regulator of immune‐cell fitness and tumor immune responses [21, 22]. This relevance stems from its dependence on iron‐driven lipid peroxidation and redox imbalance. On one hand, lipid homeostasis defects driven by dietary cues and intracellular signaling render CD8^+^ T cells more susceptible to ferroptosis, thereby diminishing effector function [23, 24]. On the other hand, redox imbalance similarly undermines CD8^+^ T cells function, as loss of ferroptosis‐protective and antioxidant programs compromises activation, persistence, and responsiveness to immunotherapy [25]. Together, these observations suggest that limiting ferroptosis in immune cells may represent a potential route to improve immunotherapy responsiveness [26]. However, whether microbiota‐derived metabolites can regulate macrophages' ferroptosis to activate anti‐tumor immunity remains unknown.

In this study, we identify indole‐3‐pyruvic acid (I3P) as an immunostimulatory molecule that promotes anti‐tumor immunity by suppressing macrophages' ferroptosis and sustaining CD8^+^ T cells function. Macrophage‐targeted nanoparticle further enhances the efficacy of I3P, thereby potentiating immunotherapy response in BCa.

## 2 Results

### 2.1 Intratumoral Bacterial Metabolite I3P Is Associated with Immunotherapy Response in Bladder Cancer

To investigate whether the intratumoral microbial ecosystem contributes to differential responses to immunotherapy, we first treated a spontaneous BCa mouse model with an anti‐PD‐1 antibody (Figure 1A). Hematoxylin and eosin (H&E) staining and tumor burden analysis showed that responder tumors displayed stronger immune infiltration and lower tumor burden than non‐responder tumors (Figure 1B). We next investigated the association between intratumoral microbiota and differential responses to immunotherapy in orthotopic BCa. Fluorescence in situ hybridization (FISH) analysis of BCa tissues revealed a significant difference in bacterial signals between responders and non‐responders, suggesting that intratumoral bacteria may influence immunotherapy efficacy in BCa (Figure 1C). To further characterize microbial differences, we performed 16S sequencing on tumor tissues. Multiple diversity metrics, including the Simpson index, showed no significant difference in overall microbial diversity between the two groups (Figure 1D). However, principal coordinates analysis (PCoA) based on weighted UniFrac distances revealed a partial separation in microbial composition between responders and non‐responders (Figure 1E). Consistent with this separation, taxonomic profiling at the order level showed that Rhizobiales, Pseudomonadales, Burkholderiales, and Lactobacillales dominated the microbial communities, with distinct abundance patterns between responders and non‐responders (Figure 1F). Functional enrichment analysis of microbial metabolic pathways further showed that the differential microbial signatures were significantly enriched in amino acid metabolism (Figure 1G). Notably, differential taxonomic analysis revealed a marked enrichment of Lactobacillus in the immunotherapy‐responsive group (Figure 1H). Consistent with the enrichment of amino acid metabolic pathways and the known role of Lactobacillus in tryptophan metabolism [27, 28, 29], we next focused on tryptophan metabolism and profiled its major metabolites using targeted metabolomics (Figure S1A). Tryptophan levels were modestly lower in responders than in non‐responders, although the difference did not reach statistical significance (Figure 1I). Focusing on the major downstream tryptophan catabolites, we found that only I3P was significantly increased in the responder group, whereas kynurenine and hydroxytryptophan showed no significant difference between the two groups (Figure 1J). Metabolite‐to‐tryptophan ratios revealed a selective increase in I3P relative to tryptophan in responders, suggesting preferential activation of I3P production in immunotherapy‐responsive BCa (Figure S1B). To determine whether this metabolic difference was derived from intratumoral bacteria, mice were treated with a broad‐spectrum triple‐antibiotic regimen to deplete host‐associated microbiota before immunotherapy, followed by quantitative mass spectrometry analysis of tryptophan metabolites. Under bacterial depletion, the difference in I3P levels between responders and non‐responders was abolished (Figure 1K), indicating that I3P in BCa was mainly derived from the tumor‐associated microbiota.

**FIGURE 1:**
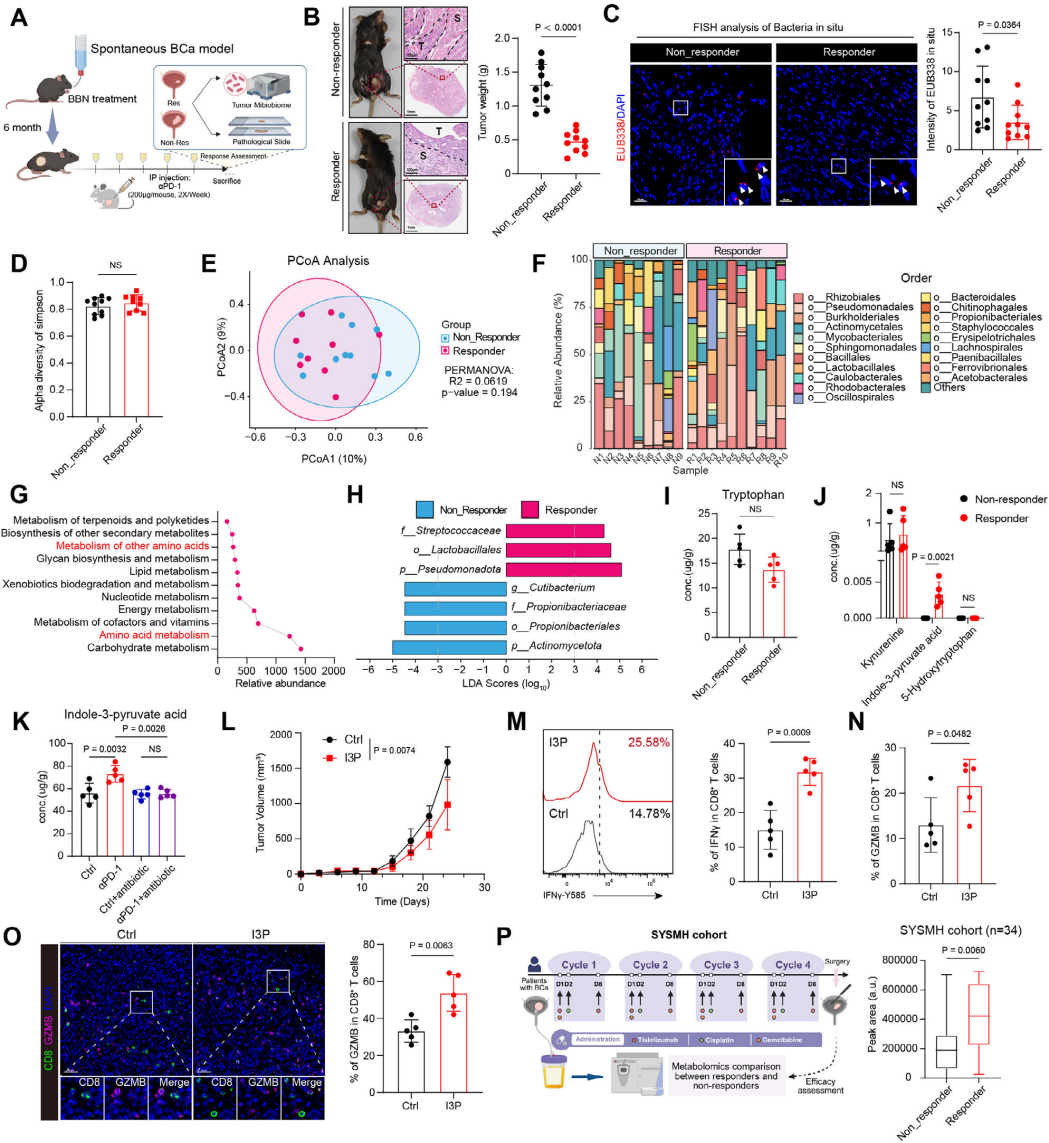
Indole‐3‐pyruvic acid is associated with immunotherapy efficacy in bladder cancer. (A) Schematic of the spontaneous BCa model for immunotherapy. (B) Representative H&E images and tumor weight quantification of spontaneous BCa tissues with distinct responses to immunotherapy. (C) FISH images and quantification of bacterial signaling in BCa tissues from distinct groups. (D) Simpson index showing α‐diversity in responder and non‐responder groups. (E) PCoA of β‐diversity based on weighted UniFrac distance. (F) Taxonomic composition of microbial communities at the order level in responder and non‐responder groups. (G) KEGG analysis of intratumoral microbiota between different groups based on 16S rRNA sequencing. (H) LDA scores of differentially abundant taxa between groups with distinct therapeutic outcomes. (I) Targeted metabolomic analysis showing tryptophan levels in non‐responder and responder BCa samples. (J) Targeted tryptophan metabolomic showing differences in the major downstream metabolites of tryptophan. (K) Metabolomic analysis of I3P in the antibiotic‐treated and control groups. (L) Tumor growth curves of mice with or without I3P treatment (50 mg kg^−1^, oral gavage, daily). (M) Representative flow cytometry plots and quantification of IFNγ in CD8^+^ T cells. (N) Flow cytometric analysis of GZMB in CD8^+^ T cells. (O) Representative mIF images and quantification of GZMB in CD8^+^ T cells in BCa from mice with or without I3P treatment. (P) Retrospective metabolomic analysis was performed with pretreatment urine samples from BCa patients receiving chemotherapy plus anti‐PD‐1 immunotherapy at SYSMH. Patients were divided into non‐responders (SD/PD, *n* = 17) and responders (CR/PR, *n* = 17) according to treatment response. BCa, Bladder cancer; CR, complete response; FISH, Fluorescence in situ hybridization; I3P, Indole‐3‐pyruvic acid; mIF, Multiplex immunofluorescence; PCoA, Principal coordinates analysis; PD, progressive disease; PR, partial response; SD, stable disease.

To examine the functional impact of I3P on BCa, we established a subcutaneous BCa model and treated mice with I3P or a vehicle control via oral gavage for 18 days (Figure S1C,D). Tumor growth analysis showed that I3P treatment significantly reduced tumor burden compared with the control group, supporting an anti‐tumor effect of I3P (Figure 1L; Figure S1E). Flow cytometry further showed that I3P increased the proportion of GZMB^+^ and IFN‐γ^+^ CD8^+^ T cells, consistent with enhanced cytotoxic effector function (Figure 1M,N). In line with this, multiplex immunofluorescence (mIF) analysis of tumor tissues confirmed that I3P was associated with a more activated anti‐tumor immune microenvironment (Figure 1O). Further analysis addressed whether I3P directly affects malignant phenotypes of BCa cells. MB49 cells were treated with I3P in vitro, and I3P treatment did not significantly alter colony formation, transwell migration, or wound healing (Figure S1F–H), suggesting minimal direct effects of I3P on cancer cells.

We then evaluated the association between I3P and ICB response in BCa patients from a BCa cohort (SYSMH). Metabolomic profiling revealed that responders had significantly higher urinary levels of I3P than non‐responders (Figure 1P; Table S1). Together with our in vivo findings, these data highlight a positive association between I3P abundance and favorable immunotherapy response in BCa.

### 2.2 I3P Exerts Anti‐Tumor Effects by Suppressing Macrophages Ferroptosis

Flow cytometric analysis of BCa showed that immune cell activity was broadly increased in the I3P‐treated group compared with the control group (Figure 2A), prompting us to investigate whether I3P reshapes the BCa immune microenvironment by altering cell death programs in tumor‐infiltrating immune cells. To address this, we performed single‐cell RNA sequencing (scRNA‐seq) on CD45^+^ cells sorted from orthotopic BCa treated with I3P. Unsupervised clustering and annotation based on established marker genes identified the major cell populations, including neutrophils, macrophages, CD8^+^ T cells, CD4^+^ T cells, NK cells, dendritic cells, fibroblasts, mast cells, B cells, and plasma cells (Figure 2B; Figure S2A). UMAP visualization by treatment group, together with cell proportion analysis, showed that I3P treatment markedly increased the proportion of intratumoral macrophages (Figure S2B,C). To further define the effect of I3P on CD8^+^ T cell activation, we re‐clustered CD8^+^ T cells using established functional markers (Figure S2D,E). Relative observed‐to‐expected (R/O/E) analysis showed that only the effector CD8^+^ T cell subset displayed preferential enrichment in the I3P‐treated group (Figure 2C).

**FIGURE 2:**
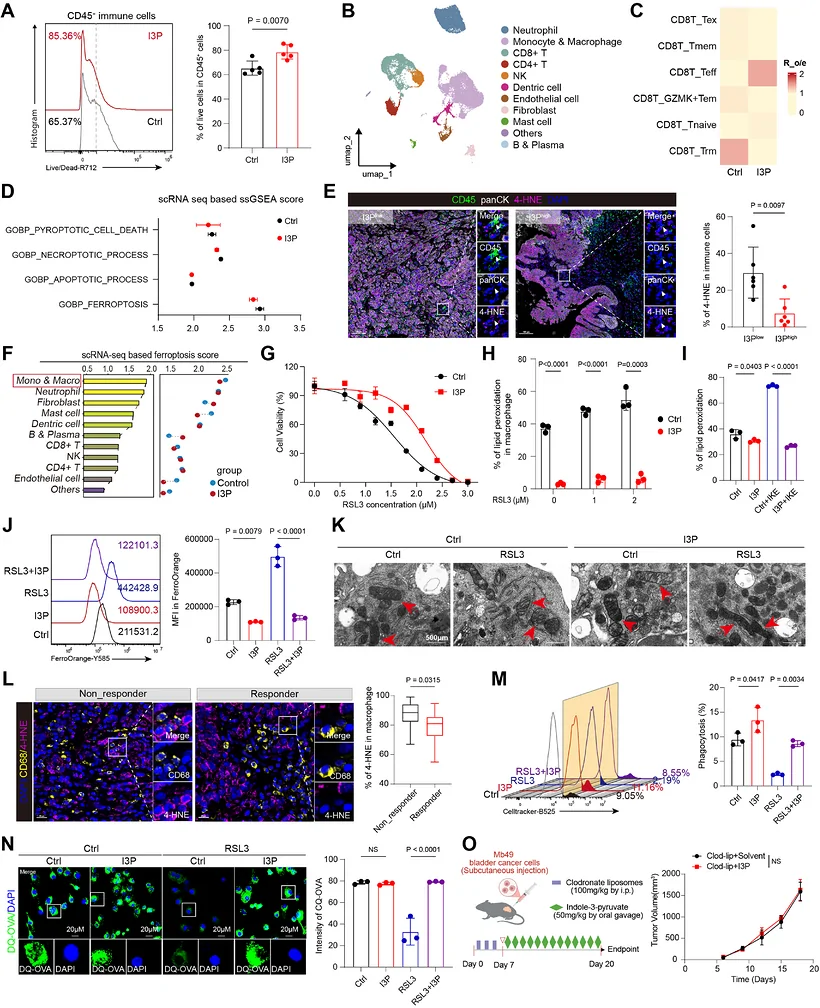
Indole‐3‐pyruvic acid activates antitumor immunity by suppressing macrophages' ferroptosis. (A) Flow cytometric analysis of living cells in CD45^+^ immune cells. (B) UMAP plot of major cell types identified by scRNA sequencing of CD45^+^ enriched BCa tissues. (C) Heatmap showing the phenotype preferences of CD8^+^ T cell subsets by Ro/e. (D) ssGSEA analysis of distinct cell death programs between groups. (E) Representative mIF images and quantification of 4‐HNE in immune cells in spontaneous BCa stratified by I3P abundance. (F) Ferroptosis pathway scores across major cell types. (G) Relative viability assay of BMDMs treated with vehicle or I3P (200 µm) in the presence of RSL3. (H) Flow cytometric analysis and quantification of lipid peroxidation in BMDMs treated with I3P (200 µm) and/or RSL3 (1 µm) for 24 h. (I) Flow cytometric analysis and quantification of lipid peroxidation in BMDMs treated with I3P (200 µm) and/or IKE (5 µm). (J) Representative flow cytometry showing Fe2^+^ uptake in BMDMs after treatment with I3P (200 µm) and/or RSL3 (1 µm). (K) Representative TEM images of mitochondria in BMDMs pretreated with I3P (200 µm) for 12 h and then exposed to RSL3 (1 µm) for 24 h; red arrows indicate mitochondrial morphological changes. (L) Representative mIF images and quantification of 4‐HNE^+^ macrophages in BCa specimens from patients receiving immunotherapy. (M) Representative flow cytometry plots and quantification of BMDM phagocytosis of BCa cells MB49 following the indicated treatment. (N) Representative confocal images and quantification of DQ‐OVA antigen presentation by BMDM. (O) Schematic of the macrophage depletion model in subcutaneous BCa in C57BL/6 mice treated with vehicle or I3P, along with tumor growth curves. BCa, Bladder cancer; BMDM, Bone marrow‐derived macrophages; I3P, Indole‐3‐pyruvic acid; mIF, Multiplex immunofluorescence; ssGSEA, single‐sample Gene Set Enrichment Analysis.

Given that I3P appeared to reshape the immune landscape, we next systematically assessed multiple forms of programmed cell death at the single‐cell level, including apoptosis, necroptosis, pyroptosis, and ferroptosis [30, 31]. Single‐sample gene set enrichment analysis (ssGSEA) showed that, among these pathways, ferroptosis signatures exhibited the most prominent change following I3P treatment (Figure 2D). mIF staining of orthotopic BCa further confirmed that I3P significantly reduced ferroptosis in tumor‐infiltrating immune cells (Figure 2E). Comparison across immune cell populations revealed that macrophages exhibited the highest ferroptosis activity among all immune subsets, and this ferroptosis signature was markedly decreased after I3P treatment (Figure 2F). To further define how I3P reshapes the macrophage compartment, we re‐analyzed macrophage subsets and found that their composition was substantially altered after treatment (Figure S3A). In particular, the Mac_Spp1 subset, which displayed high ferroptosis activity, was markedly reduced following I3P treatment, whereas the Mac_Cd86 subset was increased (Figure S3B,C). Pathway enrichment analysis further revealed that Mac_Spp1 was characterized by stronger lipid catabolic and ferroptosis‐related programs, whereas Mac_Cd86 displayed a more immune‐activated phenotype (Figure S3D).

Consistent with this observation, we tested whether I3P directly protects macrophages from ferroptosis in vitro. In bone marrow‐derived macrophages (BMDMs), I3P treatment significantly increased resistance to RSL3‐induced cell death (Figure 2G). Moreover, I3P markedly reduced lipid peroxidation in macrophages, and this effect was maintained across different concentrations of RSL3 (Figure 2H). To exclude the possibility that this effect was specific to the ferroptosis inducer, we further used IKE and observed that I3P similarly reversed IKE‐induced lipid peroxidation (Figure 2I). In addition, FerroOrange staining showed that I3P attenuated the RSL3‐induced accumulation of intracellular ferrous iron, further indicating an overall reduction of ferroptotic stress in macrophages (Figure 2J). Transmission electron microscopy (TEM) showed that RSL3‐treated macrophages exhibited typical ferroptotic morphological features, including mitochondrial shrinkage and loss of cristae, whereas I3P pretreatment largely reversed these alterations (Figure 2K). We examined whether macrophages' ferroptosis differed according to ICB response in the SYSMH cohort. mIF analysis showed a lower proportion of ferroptotic macrophages in responders than in non‐responders, supporting the clinical relevance of macrophage ferroptosis in BCa immunotherapy (Figure 2L).

We next asked whether the anti‐ferroptotic effect of I3P helps maintain macrophages' immune‐supportive functions [32], particularly phagocytosis and antigen presentation. In a macrophage–tumor cell co‐culture system, I3P significantly rescued the reduction in macrophages' phagocytic activity induced by RSL3 during 12 h of co‐culture (Figure 2M). Similarly, confocal imaging using DQ‐ovalbumin showed that RSL3 impaired macrophages' antigen processing and presentation, whereas I3P treatment markedly restored the capacity (Figure 2N). To determine whether macrophages are necessary for the anti‐tumor effects of I3P in vivo, we further performed macrophage depletion experiments. Under macrophage‐depleted conditions, I3P monotherapy no longer exerted a significant anti‐tumor effect (Figure 2O; Figure S3E,F). Collectively, these data support macrophages' ferroptosis suppression as a key mechanism underlying the anti‐tumor effect of I3P in BCa.

### 2.3 Suppression of Macrophage Ferroptosis by I3P Sustains CD8^+^ T Cell Activation

Because dying cells can communicate with the TIME during ferroptosis [33, 34], we hypothesized that suppression of macrophage ferroptosis by I3P reshapes macrophage–CD8^+^ T cell communication. CellChat analysis showed that interactions between macrophages and CD8^+^ T cells were preferentially enhanced after I3P treatment (Figure S3G). To functionally validate this axis in vitro, we cultured CD8^+^ T cells with conditioned media from macrophages pretreated with I3P and/or RSL3 for 24 h, followed by RSL3 stimulation. Conditioned media from I3P‐pretreated macrophages significantly reduced lipid peroxidation in CD8^+^ T cells, indicating that suppression of macrophages' ferroptosis by I3P alleviates ferroptotic stress in CD8^+^ T cells (Figure 3A). Consistently, confocal imaging of mitochondrial reactive oxygen species further showed that conditioned media from I3P‐treated macrophages helped maintain redox homeostasis in CD8^+^ T cells (Figure 3B). We next asked whether this reduction in oxidative stress translates into improved CD8^+^ T cell function. Indeed, conditioned media from I3P‐pretreated macrophages markedly reversed the suppression of CD8^+^ T cell effector activity induced by RSL3 (Figure 3C,D). Moreover, when activated CD8^+^ T cells were co‐cultured with labeled tumor cells for 12 h, conditioned media from I3P‐pretreated macrophages more effectively enhanced CD8^+^ T cell‐mediated tumor killing (Figure 3E).

**FIGURE 3:**
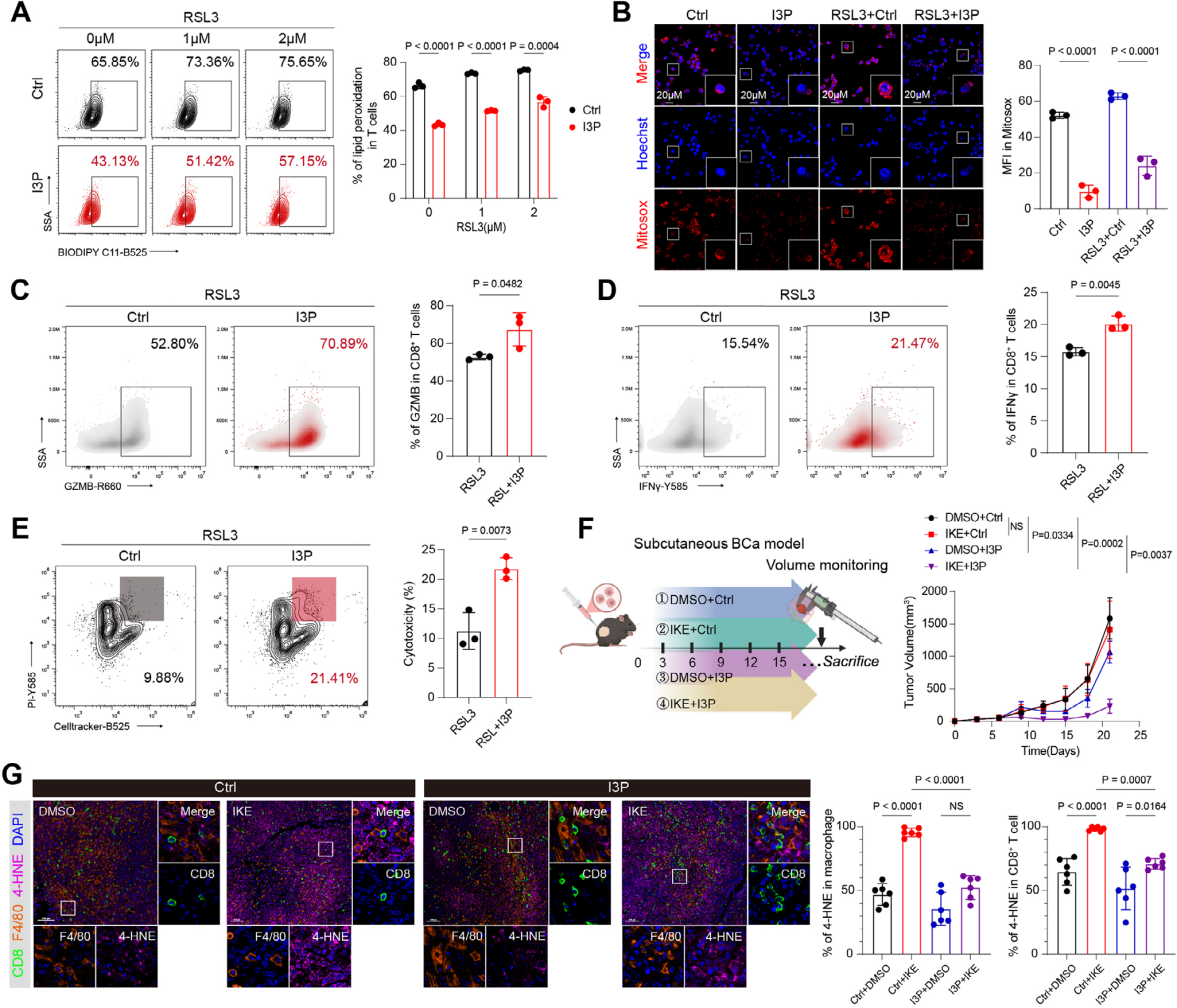
Indole‐3‐pyruvic acid enhances effector CD8^+^ T cell function. (A) Representative flow cytometry plots and quantification of lipid peroxidation in CD8^+^ T cells exposed to conditioned medium from I3P (200 µm) and/or RSL3‐treated BMDMs for 24 h, followed by 24 h of RSL3 (0.5 µm) stimulation. (B) Representative confocal images and quantification of mitochondrial ROS in CD8^+^ T cells exposed to conditioned medium from BMDMs for 24 h and then stimulated with RSL3 (0.5 µm). (C) Representative flow cytometry plots and quantification of GZMB in CD8^+^ T cells after the indicated stimulation. (D) Representative flow cytometry plots and quantification of IFNγ in CD8^+^ T cells after the indicated stimulation. (E) Representative flow cytometry plots and quantification of tumor cell killing by stimulated CD8^+^ T cells. (F) Schematic of the experimental design and tumor growth curves of subcutaneous BCa in C57BL/6 mice treated with IKE (10 mg kg^−1^, intraperitoneally, daily) and/or I3P (50 mg kg^−1^, oral gavage, daily). (G) Representative mIF images and quantitative analysis of murine BCa tissues from different treatment groups. BCa, Bladder cancer; BMDM, Bone marrow‐derived macrophages; I3P, Indole‐3‐pyruvic acid; mIF, Multiplex immunofluorescence.

To extend these findings in vivo, we further asked whether the effect of I3P on CD8^+^ T cells is macrophage‐dependent. In BCa from macrophage‐depleted mice, I3P no longer enhanced effector CD8^+^ T cell activation (Figure S3H) or reduced lipid peroxidation in CD8^+^ T cells (Figure S3I), as shown by mIF staining and flow cytometric analysis. Consistently, direct treatment of CD8^+^ T cells with I3P failed to alter lipid peroxidation or significantly affect IFN‐γ and GZMB expression, as well as CD8^+^ T cell‐mediated tumor killing (Figure S3J–L). These findings jointly indicate that I3P supports CD8^+^ T cell function indirectly through macrophages.

We next examined whether macrophages and CD8^+^ T cells exhibit coordinated ferroptotic changes in vivo under ferroptotic stress. Mice bearing BCa were treated with IKE and/or I3P for 18 days. Tumor growth analysis showed that I3P significantly restrained tumor growth under ferroptotic stress (Figure 3F; Figure S3M). mIF staining further showed that I3P attenuated IKE‐induced ferroptosis in macrophages, while macrophages and CD8^+^ T cells displayed similar trends across treatment groups (Figure 3G). These data support a coordinated ferroptotic response within the macrophage–CD8^+^ T cell crosstalk and further suggest that the anti‐ferroptotic effect of I3P in macrophages further promotes CD8^+^ T cell activation.

### 2.4 I3P Suppresses Macrophages Ferroptosis Through AHR

Previous studies have established AHR as a major sensor of exogenous small molecules and a critical mediator of tryptophan metabolite signaling [35, 36]. On this basis, we explored the potential interaction between I3P and AHR. Molecular docking analysis showed that I3P could bind to the predicted ligand‐binding region of AHR (Figure 4A), suggesting that AHR may mediate the effect of I3P on macrophages. In line with it, cellular thermal shift assays (CETSA) further showed that I3P markedly altered the thermal stability of AHR in macrophages (Figure 4B), consistent with target engagement of AHR by I3P in macrophages. Given that macrophages emerged as the major cellular target of I3P in our previous analyses, we next examined whether AHR was preferentially expressed in this population. scRNA‐seq showed that Ahr expression was highest in macrophages among immune cells (Figure S4A). Consistent with this, macrophage re‐clustering further showed that the subsets with higher Ahr expression displayed less ferroptosis enrichment (Figure S3B). mIF staining of spontaneous BCa further confirmed that AHR was significantly enriched in tumor‐associated macrophages compared with other immune cells (Figure S4B). Together, these findings suggest that the downstream response to I3P is closely linked to AHR‐expressing macrophages in BCa.

**FIGURE 4:**
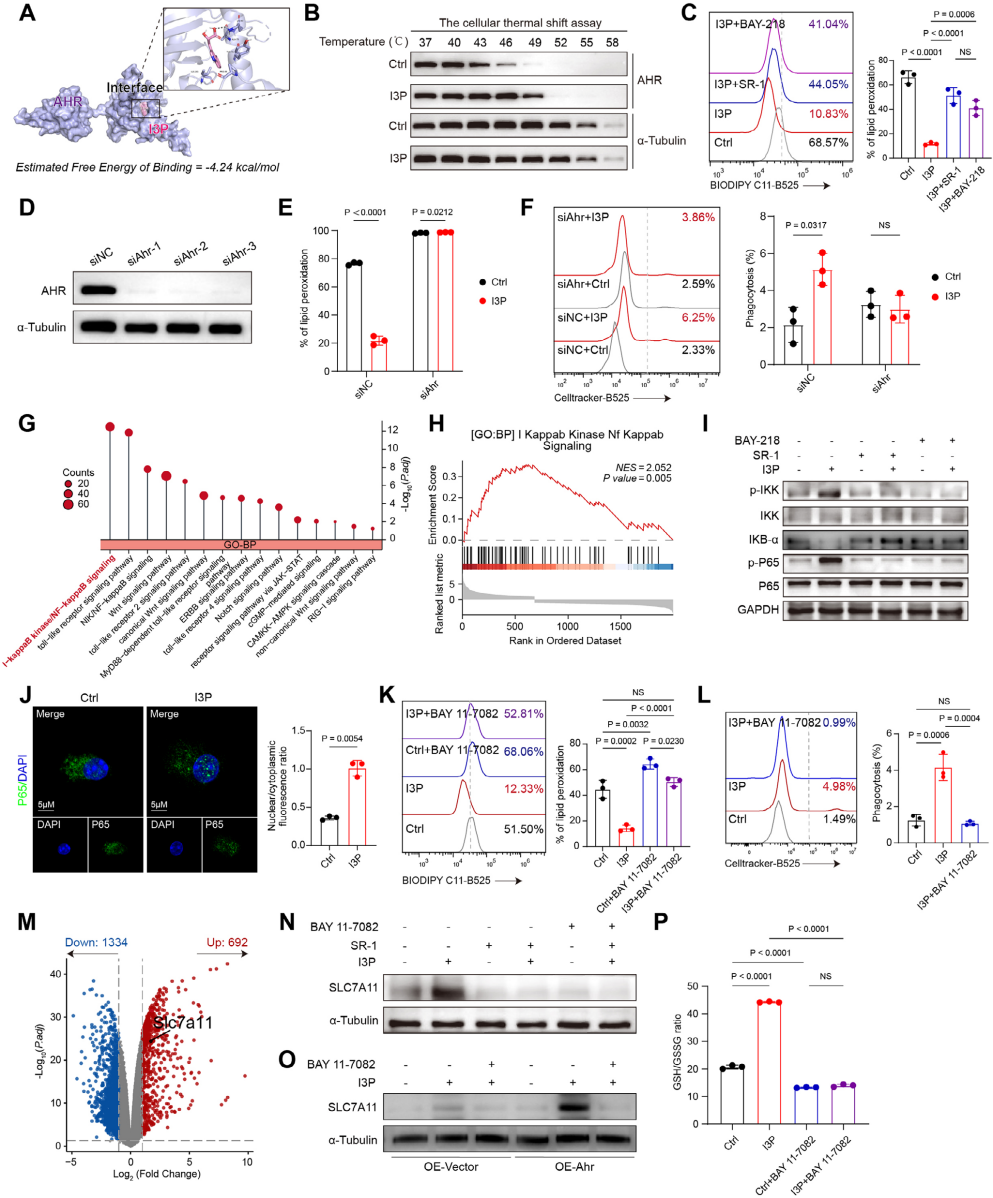
Indole‐3‐pyruvic acid activates AHR‐mediated IKK/IκB/P65 signaling to increase SLC7A11 expression. (A) The representative interaction of the I3P/AHR complex by molecular docking. (B) The cellular thermal shift assay of I3P (200 µm) or vehicle‐treated BMDM. **(C)** Representative flow cytometry plots and quantification of lipid peroxidation in BMDMs treated with I3P (200 µm) with or without AHR inhibitor (10 µm BAY218; 10 µm SR‐1). (D) Western blotting validation of Ahr knockdown efficiency in siAhr‐transfected BMDMs. (E) Representative flow cytometry plots and quantification of lipid peroxidation in siAhr‐transfected BMDMs. (F) Representative flow cytometry plots and quantification of tumor cell phagocytosis by siAhr‐transfected BMDMs. (G) Lollipop chart of GO enrichment involving the major signaling pathways in BMDM with or without I3P treatment (200 µm). (H) GSEA of the IκB kinase/NF‐κB Signaling pathway in BMDM treated with or without I3P (200 µm). (I) Western blotting analysis of crucial proteins in the canonical NF‐κB signaling pathway of the indicated BMDM. (J) Representative confocal images indicate the translocation of p65 in BMDM with or without I3P treatment (200 µm). (K) Representative flow cytometry plots and quantification of lipid peroxidation in BMDMs treated with I3P with or without NF‐κB inhibitor (5 µm BAY 11–7082). (L) Representative flow cytometry analysis of tumor cell phagocytosis by BMDMs treated with I3P with or without NF‐κB inhibitor (5 µM BAY 11–7082). (M) Volcano plot of differentially expressed genes in BMDM with or without I3P (200 µm) treatment. (*p* < 0.05, |log2 FC| ≥ 1). (N) Western blotting analysis of SLC7A11 expression in BMDM of the indicated treatment. (O) Western blotting analysis of SLC7A11 expression in BMDM after mRNA‐induced AHR overexpression following indicated stimulation. (P) GSH/GSSG levels were examined in BMDM with or without I3P treatment (200 µm). AHR, Aryl hydrocarbon receptor; BCa, Bladder cancer; BMDM, Bone marrow‐derived macrophages; GSEA, Gene Set Enrichment Analysis; I3P, Indole‐3‐pyruvic acid.

We then tested whether AHR is required for the anti‐ferroptotic effect of I3P in macrophages. Pharmacologic inhibition of AHR markedly restricted the ability of I3P to suppress ferroptosis (Figure 4C). Consistent with this, siRNA‐mediated silencing of endogenous Ahr similarly abolished the anti‐ferroptotic effect of I3P (Figure 4D,E). Functionally, AHR blockade also impaired the ability of I3P to restore macrophages' phagocytic activity under ferroptotic stress (Figure 4F). Together, these findings establish AHR as a key mediator through which I3P suppresses macrophages' ferroptosis and preserves macrophages' function.

### 2.5 I3P Activates AHR‐Dependent NF‐κB Signaling to Promote SLC7A11 Transcription

To define the mechanism of AHR activation by I3P in macrophages, we performed transcriptomic profiling of I3P‐treated macrophages. Gene ontology (GO) enrichment analysis identified the canonical NF‐κB signaling pathway as one of the most prominently enriched pathways following I3P treatment (Figure 4G), which was further supported by gene set enrichment analysis (GSEA) (Figure 4H). Consistent with these findings, Western blotting analysis showed that I3P markedly increased the phosphorylation levels of IKK and p65, indicating activation of the canonical NF‐κB pathway (Figure 4I). This effect was abolished by pharmacologic inhibition of AHR, indicating that NF‐κB activation occurs downstream of AHR signaling. In line with it, confocal imaging revealed enhanced nuclear translocation of p65 in macrophages upon I3P treatment, further supporting activation of NF‐κB–mediated transcriptional regulation (Figure 4J). To determine whether NF‐κB activation is functionally required for the anti‐ferroptotic effect of I3P, we inhibited NF‐κB signaling using BAY 11–7082. NF‐κB blockade significantly attenuated the protective effect of I3P against ferroptosis in macrophages (Figure 4K) and impaired the ability of I3P to restore macrophages' phagocytic activity toward tumor cells (Figure 4L). Consistently, in vivo administration of BAY 11–7082 markedly abrogated the anti‐tumor effects mediated by I3P (Figure S4C). Flow cytometric analysis showed that BAY 11–7082 reversed the I3P‐induced suppression of lipid peroxidation in both macrophages and CD8^+^ T cells (Figure S4D,E). Further analysis of CD8^+^ T cell effector function revealed that NF‐κB inhibition significantly reduced the expression of GZMB and IFN‐γ (Figure S4F). These findings identify NF‐κB as a critical downstream effector linking I3P‐induced ferroptosis suppression to preserved macrophage function.

We next examined whether NF‐κB activation by I3P is linked to transcriptional induction of key ferroptosis regulators. Among ferroptosis‐related differentially expressed genes, Slc7a11 was one of the most strongly upregulated transcripts in I3P‐treated macrophages (Figure 4M). Western blotting analysis further confirmed that I3P increased SLC7A11 expression, whereas inhibition of either AHR or NF‐κB abolished this induction (Figure 4N). Notably, AHR overexpression further enhanced I3P‐induced SLC7A11 upregulation in macrophages, yet pharmacologic inhibition of NF‐κB consistently abolished this effect, supporting that NF‐κB is required downstream of AHR for SLC7A11 induction (Figure 4O). Further analysis of SLC7A11 function in macrophages ferroptosis showed that SLC7A11 knockdown in BMDMs markedly attenuated the anti‐ferroptotic effect of I3P, supporting SLC7A11 as a key downstream mediator of I3P activity (Figure S4G,H). Functionally, I3P treatment markedly increased the GSH/GSSG ratio in macrophages, whereas NF‐κB inhibition reversed this effect (Figure 4P), linking the AHR–NF‐κB–SLC7A11 axis to preservation of redox homeostasis. Together, these findings establish that I3P activates an AHR–NF‐κB–SLC7A11 signaling cascade to suppress macrophages' ferroptosis.

### 2.6 Macrophage‐Targeted Nanodelivery Potentiates the Immunoregulatory Activity of I3P

The identification of macrophages as the key cellular target of I3P prompted us to test whether macrophage‐directed delivery could amplify its biological effects. We therefore engineered a macrophage‐targeted liposomal nanodelivery platform for I3P [37, 38] (Figure 5A). Dynamic light scattering analysis showed that the resulting nanoparticles had an average diameter of 140 nm with a polydispersity index (PDI) of 0.17, indicating a uniform size distribution (Figure 5B). The zeta potential was −12.63 ± 4.65 mV, consistent with favorable colloidal stability. In addition, the encapsulation efficiency reached 56.5%, with a drug loading efficiency of 11.30%. Release profiling further showed that Nano@I3P exhibited a sustained‐release profile under physiological conditions (Figure 5C). We next asked whether Nano@I3P could be preferentially taken up by macrophages. Single‐cell suspensions prepared from fresh BCa were incubated with Nano@I3P for 2 h, followed by assessment of nanoparticle uptake across major immune cell populations. Flow cytometric analysis further confirmed the preferential uptake of Nano@I3P by tumor‐associated macrophages compared with other immune cell populations (Figure 5D).

**FIGURE 5:**
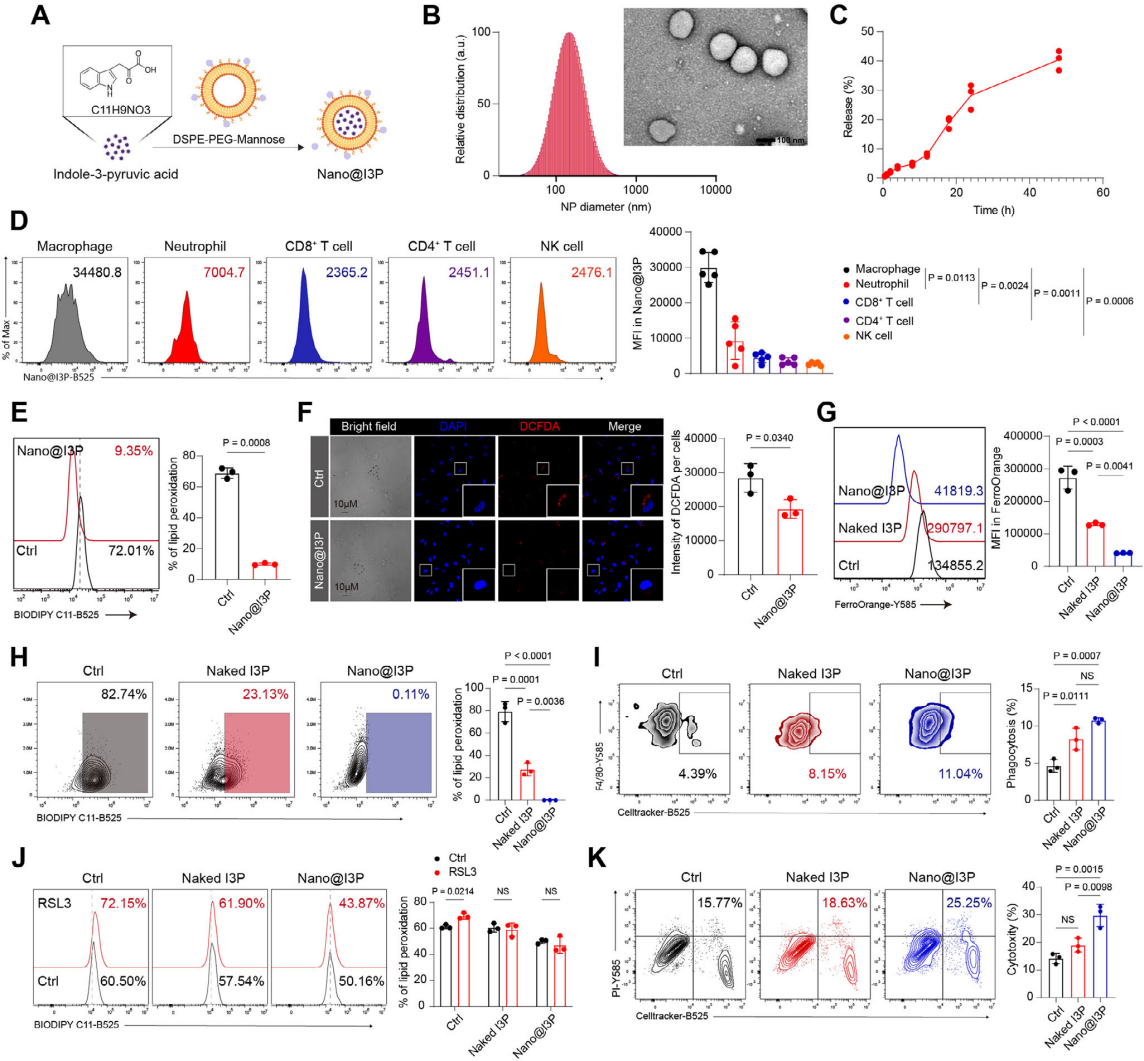
Macrophage‐targeted nanodelivery of indole‐3‐pyruvic acid enhances antitumor immunity. (A) Schematic of the liposomal nanoparticle platform composed of DSPE‐PEG‐Mannose and I3P. (B) Size distribution and morphology of Nano@I3P in aqueous solution. (C) Cumulative release of Nano@I3P in aqueous solution. (D) Representative flow cytometry plots and quantification of Nano@I3P uptake by immune cells in BCa‐derived single‐cell suspensions. (E) Representative flow cytometry plots and quantification of lipid peroxidation in Nano@I3P (20 µg mL^−1^) ‐treated BMDMs. (F) Representative confocal analysis of ROS levels in Nano@I3P (20 µg mL^−1^) treated BMDMs. (G) Representative flow cytometry showing Fe2^+^ uptake in BMDMs after treatment with solvent, naked I3P (40 µg mL^−1^), or Nano@I3P (40 µg mL^−1^). (H) Representative flow cytometry of ROS levels in BMDMs treated with Nano@I3P (40 µg mL^−1^) versus naked I3P (40 µg mL^−1^). (I) Representative flow cytometry of tumor cell phagocytosis by BMDMs treated with Nano@I3P versus naked I3P. (J) Representative flow cytometry plots and quantification of ROS levels in RSL3‐stimulated CD8^+^ T cells exposed to conditioned medium from BMDMs treated with Nano@I3P (40 µg mL^−1^) or naked I3P (40 µg mL^−1^). (K) Representative flow cytometry plots and quantification of tumor cell killing by CD8^+^ T cells stimulated with conditioned medium from BMDMs treated with Nano@I3P or naked I3P. BCa, Bladder cancer; BMDM, Bone marrow‐derived macrophages; I3P, Indole‐3‐pyruvic acid.

We then examined whether nanoparticle‐mediated delivery enhances the anti‐ferroptotic activity of I3P in macrophages. In vitro treatment of macrophages with Nano@I3P markedly reduced lipid peroxidation and mitochondrial reactive oxygen species (Figure 5E,F). Consistently, intracellular ferrous iron levels were significantly suppressed following Nano@I3P treatment (Figure 5G). Compared with free I3P at the same concentration, Nano@I3P more effectively suppressed lipid peroxidation as determined by flow cytometry (Figure 5H). This stronger anti‐ferroptotic effect was accompanied by a more efficient restoration of macrophages' phagocytic activity under ferroptotic stress in co‐culture assays with tumor cells (Figure 5I). The enhanced protection of macrophages by Nano@I3P led us to ask whether this effect could be translated into stronger support of effector CD8^+^ T cells. Conditioned media from macrophages treated with Nano@I3P or free I3P were used to stimulate CD8^+^ T cells prior to RSL3 exposure. Compared with free I3P, Nano@I3P more effectively reduced lipid peroxidation in CD8^+^ T cells (Figure 5J), and this reduction in ferroptotic stress was accompanied by a more robust restoration of CD8^+^ T cell cytotoxicity (Figure 5K). Together, these findings show that Nano@I3P more effectively potentiates the anti‐ferroptotic and immunoregulatory activity of I3P.

### 2.7 Nanoparticle‐mediated Delivery of I3P Overcomes Resistance to Anti‐PD‐1 Therapy

Following the enhanced activity observed in vitro, we next evaluated whether Nano@I3P, compared with free I3P, exhibits improved tumor accumulation and therapeutic efficacy in vivo. We first assessed its biodistribution following intravenous administration in tumor‐bearing mice. Ex vivo imaging of major organs at 6 h post‐injection showed that Nano@I3P exhibited markedly higher tumor accumulation than free I3P (Figure 6A). We next tested the therapeutic potential of Nano@I3P in an immunotherapy‐resistant BCa model established through three successive rounds of in vivo immune checkpoint blockade (Figure 6B; Figure S5A). mIF staining confirmed an immune‐suppressed phenotype in ICB‐resistant MB49‐derived BCa with reduced CD8^+^ T cell infiltration and impaired cytotoxic function. (Figure S5B). Treatment with Nano@I3P alone significantly reduced tumor burden compared with controls, while combination therapy with Nano@I3P and anti–PD‐1 further enhanced tumor suppression and overcame resistance to immune checkpoint blockade (Figure 6C–E). No significant differences in body weight were observed among treatment groups during the treatment period (Figure S5C). In addition, histological analysis revealed no obvious tissue damage in major organs following Nano@I3P administration, indicating a favorable safety profile (Figure S5D). Flow cytometric analysis showed that Nano@I3P, particularly in combination with anti–PD‐1, increased the overall infiltration of immune cells within the TIME (Figure 6F). Notably, the proportion of CD8^+^ T cells was significantly elevated in both the Nano@I3P and combination groups, with the most pronounced increase observed under combination treatment (Figure 6G), suggesting enhanced anti‐tumor immune activation. Consistent with these findings, mIF staining revealed a marked reduction in ferroptotic stress following Nano@I3P treatment in macrophages and CD8^+^ T cells, particularly in the combination group (Figure 6H).

**FIGURE 6:**
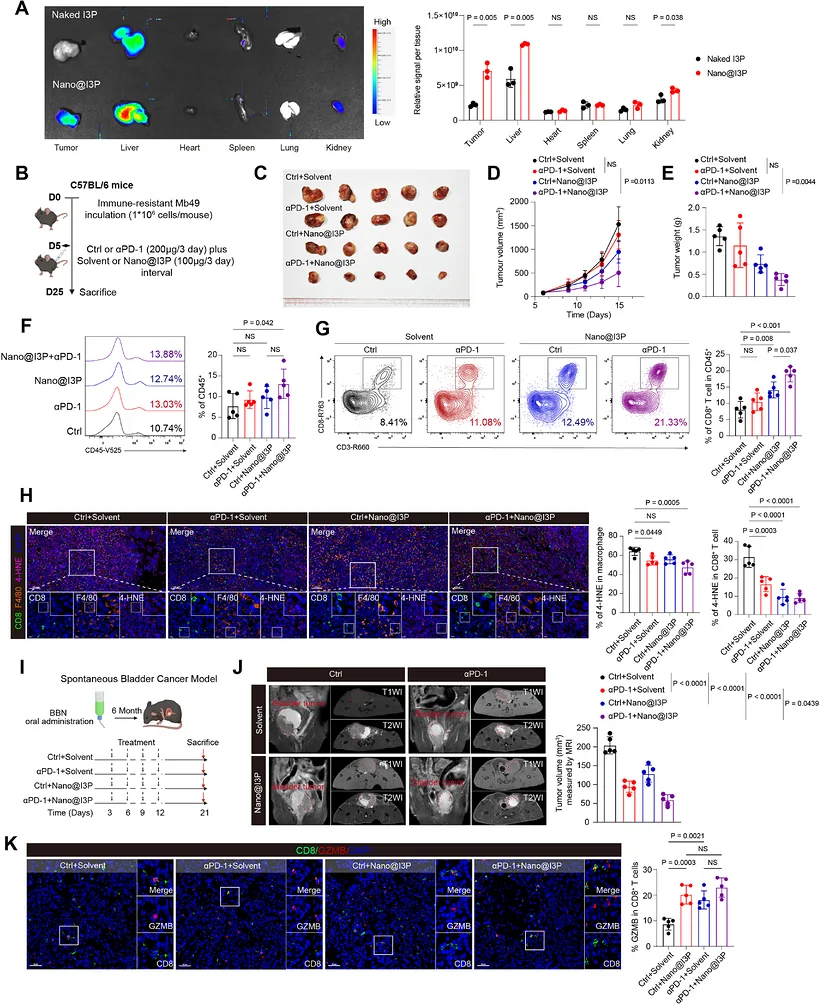
Nano@I3P overcomes immunotherapy resistance in bladder cancer. (A) Representative fluorescence images and quantification of the bio‐distribution of naked I3P and Nano@I3P in tumors and major organs at 6 h after tail vein injection. (B) Schematic of the immunotherapy‐resistant subcutaneous BCa model in C57BL/6 mice treated with Nano@I3P (5 mg kg^−1^) and/or αPD‐1 (10 mg kg^−1^). (C–E) Representative gross images (C), tumor growth curves (D), and tumor weight (E) of BCa from different groups. (F) Representative flow cytometry plots and quantification of immune cell infiltration in BCa from different groups. (G) Representative flow cytometry plots and quantification of CD8^+^ T cell proportions among tumor‐infiltrating immune cells. (H) Representative mIF images and quantitative analysis of BCa from the indicated treatment groups. (I) Schematic of the BBN‐induced spontaneous BCa model in C57BL/6 mice treated with Nano@I3P (5 mg kg^−1^) and/or αPD‐1 (10 mg kg^−1^). (J) Representative MRI images and quantification of tumor volume in the spontaneous BCa model. (K) Representative mIF images and quantitative analysis of BCa from the indicated treatment groups. BBN, N‐butyl‐N‐(4‐hydroxybutyl) nitrosamine; BCa, Bladder cancer; I3P, Indole‐3‐pyruvic acid; mIF, Multiplex immunofluorescence.

To further extend these findings, we employed a BBN‐induced spontaneous BCa model (Figure 6I). Tumor burden was monitored by MRI after 18 days of treatment. Both Nano@I3P and anti–PD‐1 monotherapy exerted anti‐tumor effects, whereas the combination therapy achieved the most substantial tumor suppression (Figure 6J). mIF staining further confirmed that Nano@I3P enhanced immune activation in the orthotopic BCa (Figure 6K). Together, these findings demonstrate that Nano@I3P improves tumor‐targeted delivery of I3P, enhances its in vivo efficacy, and effectively overcomes immunotherapy resistance in BCa.

## 3 Discussion

This study uncovers a microbiota‐related immune pathway with both mechanistic insight and translational relevance in BCa. We identify I3P as an immunostimulatory molecule that promotes anti‐tumor immunity and establish suppression of macrophages' ferroptosis as a central mechanism underlying its immunostimulatory activity. Importantly, macrophage‐targeted nanodelivery further improves its efficacy and capacity to sensitize BCa to ICB.

Accumulating studies link tumor microbiota to responses to cancer immunotherapy [39, 40]. However, their precise contribution remains incompletely understood, in part because microbiota depletion can also impair ICB efficacy [41]. Clinical evidence from the PURE‐01 study further supports this notion, showing that antibiotic use is associated with lower complete response rates and reduced recurrence‐free survival in patients with BCa [42]. These observations raise the possibility that distinct microbiota communities may exert beneficial immunomodulatory effects in BCa. In this context, we identified the Lactobacillus‐associated metabolite I3P as enriched in ICB‐responsive tumors. Previously, microbiota‐derived metabolites have been shown to directly shape tumor immunity. For example, indole‐3‐propionic acid can induce apoptosis of Th1 and Th17 cells [43], whereas trans‐3‐indoleacrylic acid acts as an AHR ligand to promote ferroptosis resistance in tumor cells through FSP1‐dependent pathways [44]. Here, our in vitro and in vivo experiments demonstrated that I3P reduced lipid peroxidation and ferroptosis‐associated cell death in macrophages while preserving phagocytic and antigen‐presenting capacity under ferroptotic stress.

Ferroptosis can propagate through reactive oxygen species–driven signaling and influence neighboring cells [33]. Recent work has shown that Galectin‐13 secreted by ferroptotic cells can bind CD44 and inhibit membrane localization of SLC7A11 in adjacent cells, thereby amplifying ferroptotic stress [45]. Our data showed that suppression of macrophages' ferroptosis by I3P is accompanied by reduced ferroptotic stress and enhanced effector function in CD8^+^ T cells, underscoring the broader immunologic significance of I3P‐mediated control of macrophages' ferroptosis. AHR is a major sensor of microbiota‐derived indole metabolites and an important regulator of macrophage function in the TIME [36, 46]. Previous studies have linked AHR activity to immunosuppressive programs, highlighting the context‐dependent output of AHR signaling in tumor immunity [15]. In the present study, AHR was preferentially enriched in tumor‐associated macrophages, and pharmacologic inhibition of AHR markedly abrogated both the anti‐ferroptotic effect of I3P and its ability to restore macrophages' phagocytic function under ferroptotic stress. Mechanistically, we identified canonical NF‐κB signaling as a key downstream pathway engaged by I3P‐AHR signaling and established SLC7A11 as a critical target linking this axis to preservation of macrophage redox homeostasis. These findings extend current understanding of macrophages' AHR biology by showing that, in BCa, I3P‐directed AHR signaling instead activates an AHR‐NF‐κB‐SLC7A11 program under immunotherapy.

Directing macrophages toward an immune‐supportive state has emerged as a promising therapeutic strategy [47]. However, effective therapeutic reprogramming of TAMs remains difficult because of their broad functional diversity and continuous adaptation to the TIME [48]. In this context, metabolic modulation may provide a tractable route for more durable TAM reprogramming. To maximize the efficacy of I3P, we developed a liposomal nanodelivery system for preferential targeting of tumor‐associated macrophages and enhanced its immunotherapy‐sensitizing activity [38]. This nanoparticle formulation was preferentially taken up by macrophages and more effectively suppressed macrophages' ferroptotic stress than naked I3P, both in vitro and in vivo. Consistently, Nano@I3P better preserved macrophage‐dependent support of CD8^+^ T cell function. In BCa models, Nano@I3P accumulated efficiently in tumors, enhanced anti‐tumor activity, and showed robust synergy with anti‐PD‐1 therapy without apparent toxicity.

Several limitations of this study should be noted. First, although our data support microbial metabolism as a major source of intratumoral I3P, the specific microbial species and metabolic pathways responsible for its production remain to be defined. Second, while our findings identify macrophages as the principal functional target of I3P, the contribution of additional cellular components within the TIME warrants further investigation. Finally, although Nano@I3P shows encouraging efficacy and safety in preclinical models, its translational potential requires further evaluation in clinical settings.

## 4 Conclusion

In summary, we identify I3P as a microbiota‐derived regulator of anti‐tumor immunity in BCa. By suppressing macrophages' ferroptosis through an AHR–NF‐κB–SLC7A11 axis, I3P sustains CD8^+^ T cell effector function and enhances immunotherapy response. Liposomal delivery of I3P further supports its potential as a strategy for immunotherapy sensitization in BCa.

## 5 Experimental Section

### 5.1 Patient Samples

This research received approval from the Ethics Committees of Sun Yat‐sen Memorial Hospital, Sun Yat‐sen University (NO. SYSKY‐2026‐360‐01) and was carried out in compliance with established ethical guidelines. Samples from 34 BCa patients who received chemotherapy and anti‐PD‐1 immunotherapy were collected at Sun Yat‐sen Memorial Hospital, Sun Yat‐sen University (SYSMH cohort). All patients had been diagnosed independently by three pathologists. Written informed consent was obtained from all patients before sample collection.

### 5.2 Cell Line

The MB49 murine bladder cancer cell line was obtained from Millipore (Merck Millipore, Billerica, MA, USA, Cat#SCC148).

### 5.3 Primary Cell Isolation

Bone marrow‐derived macrophages (BMDMs) were isolated and cultured following established protocols. Femurs and tibias were harvested under sterile conditions, and bone marrow cells were flushed out with sterile PBS and filtered through a 70‐µm cell strainer. After centrifugation (400 × *g*, 5 min, 4°C), red blood cells were removed using lysis buffer. The remaining cells were cultured in complete DMEM (Thermo Scientific, Cat#C11995500BT) with recombinant murine M‐CSF (20 ng mL^−1^) (Sino Biological, Cat#51112‐MNAH). On day 3, half of the medium was replaced with fresh medium containing M‐CSF. On day 6, adherent cells were collected as differentiated BMDMs and verified by flow cytometry. Mouse CD8^+^ T cells were isolated from fresh spleens using the MojoSort Mouse CD8 T Cell Isolation Kit (BioLegend, Cat#480007) according to the manufacturer's instructions. Briefly, spleens were passed through a 70 µm cell strainer to prepare single‐cell suspensions, followed by red blood cell lysis. Cells were incubated with a biotin‐antibody cocktail against non‐CD8^+^ cells for 15 min and then with anti‐biotin magnetic beads for 15 min. CD8^+^ T cells were purified by magnetic separation and cultured in complete RPMI‐1640 (Thermo Scientific, Cat#C11875500BT) with recombinant mouse IL‐2 (100 U mL^−1^) (Thermo Scientific, Cat#212‐12) activated with anti‐CD3 (1 µg mL^−1^) (BioGems, Cat#05112‐25) and anti‐CD28 (1 µg mL^−1^) (BioGems, Cat#10312‐25) antibodies.

### 5.4 Subcutaneous BCa Mouse Model

C57BL/6 mice (4‐5 weeks old, weighing 18–20 g) were obtained from BesTest Bio‐Tech Co. Ltd (Zhuhai, China). MB49 cells (1 × 10^6^ cells) were resuspended in DMEM (100 µL) without FBS and injected subcutaneously into the right flank of the mice. After reaching the experimental endpoint, the tumor was cut open, weighed, and used for pathological and mechanistic studies. The animal experiments were approved by the Animal Ethical and Welfare Committee and were performed following the institutional guidelines. All research protocols were approved by the Ethics Committee (TOP‐1PZ‐GZ250711).

### 5.5 BBN‐Induced Spontaneous BCa Mouse Model

Bladder tumors were induced using N‐butyl‐N‐(4‐hydroxybutyl) nitrosamine (Sigma‐Aldrich, Cat#B8061) dissolved in drinking water (0.05%, w/v). Mice received BBN‐supplemented drinking water for a total duration of 6 months. Body weights and water consumption were monitored weekly to assess general health and ensure consistent BBN intake. Animal trials were authorized by the Animal Ethics and Welfare Committee and conducted in compliance with institutional regulations.

### 5.6 Single‐Cell RNA Sequencing

Fresh bladder cancer tissues were dissociated to obtain a single‐cell suspension and subsequently subjected to scRNA‐seq using a Novaseq 6000 platform (Illumina). Cellular suspensions were processed using a 10X Genomics GemCode Single Cell Instrument following the manufacturer's protocol with modifications. Libraries were prepared and sequenced from the resulting complementary DNAs (cDNAs) using Chromium Next GEM Single Cell 5’ Kits (v2.0). Briefly, single cells were partitioned into GEMs, where poly(A) mRNAs were reverse‐transcribed into barcoded cDNAs containing cell‐specific barcodes and UMIs. The resulting cDNAs were amplified via PCR for library preparation.

### 5.7 Bulk RNA‐Seq and GO Analysis

Total RNA was extracted from macrophages immediately lysed with TRIzol (Thermo Scientific, Cat#A33251). A KCTM Digital mRNA Library Prep Kit (Seqhealth Tech, Cat#NR084‐01) was used to construct RNA sequencing libraries following the manufacturer's instructions. The enriched libraries were quantified and sequenced on a DNBSEQ‐T7 platform (MGI) using the PE150 sequencing mode to generate paired‐end reads. Gene ontology (GO) analysis for differentially expressed genes was implemented with a *p*‐value cutoff of 0.05 to determine a statistically significant enrichment.

### 5.8 Gene Set Enrichment Analysis (GSEA)

GSEA was performed using the clusterProfiler R package, followed by differential expression analysis. The reference gene sets were downloaded from Msigdb (https:// www. gsea‐ msigdb. org/ gsea/ msigdb).

### 5.9 DNA Extraction and 16S Sequencing

For 16S rRNA sequencing and data analysis, fresh bladder samples were collected and snap‐frozen at −80°C. Microbial DNA was extracted from the tissue samples using cetyltrimethylammonium bromide. The five variable regions (V2, V3, V5, V6, and V8) of the bacterial 16S rRNA gene were amplified through PCR using region‐specific primer pairs, and the products were purified and recovered. The recovered products were then subjected to fluorescence quantification. The size and quantity of the amplicon library were assessed and sequenced on the NovaSeq PE250 platform.

### 5.10 Metabolomics

For tissue‐targeted metabolomics, frozen samples were weighed, lyophilized, and homogenized in a 2 mL tube with a 5 mm tungsten bead (65 Hz, 1 min). Metabolites were extracted with 1 mL of pre‑cooled methanol/acetonitrile/water (2:2:1, v/v/v) under ultrasonication in an ice bath for 1 h, incubated at −20°C for 1 h, and centrifuged (14,000 × *g*, 4°C, 20 min). The supernatant was vacuum‑dried, reconstituted in 50% acetonitrile, filtered (0.22 µm), and analyzed by LC‑MS/MS in multiple reaction monitoring (MRM) mode. For urine untargeted metabolomics, samples were thawed on ice and centrifuged (14 000 × *g*, 4°C, 10 min). A 100‑µL aliquot was mixed with cold methanol/acetonitrile (400 µL, 1:1, v/v), sonicated for 30 min in an ice bath, incubated at –20°C for 1 h, and centrifuged (14 000 × *g*, 4°C, 15 min). The supernatant was dried under vacuum, reconstituted in 50% acetonitrile, filtered, and analyzed.

### 5.11 Magnetic Resonance Imaging (MRI) of Spontaneous BCa Mouse Model

MRI was performed on a 9.4 T small‐animal imaging system (Bruker Biospec, Billerica, MA, USA) to evaluate the volume of spontaneous BCa. Before the experiment, anesthesia was induced, with the respiratory rate maintained at ∼60 breaths/min. Mice were carefully positioned prone in a head‐first orientation in the MRI machine at the Fifth Affiliated Hospital, Sun Yat‐sen University, to ensure optimal imaging. High‐resolution T2‐weighted and T1‐weighted MRI sequences were acquired for both transverse and coronal sections. Imaging was finally conducted to assess the response to different interventions. The images were analyzed using the Bee DICOM viewer (https://beedicom.com/).

### 5.12 Hematoxylin and Eosin (H&E) Staining

Sections were baked at 70°C for 1 h, followed by dewaxing with xylene and subsequent rehydration with varying concentrations of ethanol. Then sections were stained with Mayer's hematoxylin for 3–5 min and with eosin Y solution for 1–2 min (Biosharp, Cat#BL735A). Sections were mounted with a coverslip using a resin permanent mounting medium.

### 5.13 Multiple Immunofluorescence (mIF)

Paraffin‐embedded bladder tissue sections were deparaffinized in xylene and rehydrated through graded ethanol. Subsequently, sections were subjected to antigen retrieval. After serum blocking for 30 min at room temperature, sections were incubated with the indicated primary antibodies, followed by corresponding secondary antibodies and signal amplification reagent. TSA fluorophores (PPD520, PPD570, PPD620, and PPD690) from the PanoPANEL mIF kit (Panovue, Cat#10217100020) were used for signal detection. Nuclei were counterstained with DAPI. For immunofluorescence staining of cultured cells, cells were seeded in confocal dishes. Following treatment, cells were fixed with 4% paraformaldehyde at room temperature and blocked with bovine serum albumin (5%) for 1 h. Cells were then incubated with the indicated primary antibodies at 4°C, followed by fluorophore‐conjugated secondary antibodies for 1 h at room temperature. After DAPI counterstaining for 15 min, images were captured using a confocal microscope. The antibodies used in this study were listed in Table S2.

### 5.14 Fluorescence In Situ Hybridization (FISH) on Bladder Sections

A universal bacterial fluorescence in situ hybridization (FISH) detection kit (Focobio, Cat#D‐0015) was used according to the manufacturer's instructions. Formalin‐fixed, paraffin‐embedded bladder cancer sections were baked at 70°C for 1 h, followed by deparaffinization in xylene and rehydration through graded ethanol. To enhance probe penetration, sections were permeabilized with Proteinase K (20 µg mL^−1^ in 100 mm Tris‐HCl and 50 mm EDTA, pH 8.0) at 37°C for 15 min, rinsed in glycine/PBS (0.2%), and washed twice with PBS. A Cy3‐labeled oligonucleotide probe (Focobio, Cat#FB‐0010B) was incubated in a humidified chamber at 46°C for 16 h. After post‐hybridization washing, sections were air‐dried in the dark and counterstained with DAPI.

### 5.15 Molecular Docking

The three‐dimensional structure of AHR was obtained from the Protein Data Bank, and the chemical structure of I3P was retrieved from the PubChem database and subjected to energy minimization prior to docking. Protein preparation was performed using AutoDock Tools 1.5.2, including removal of water molecules, addition of polar hydrogen atoms, assignment of Gasteiger charges, and definition of AutoDock atom types. The receptor and ligand were then converted into PDBQT format. A grid box was defined, and docking was carried out using AutoDock Vina. The pose with the lowest binding energy was selected for further analysis according to binding affinity. The interaction between AHR and I3P was visualized using PyMOL.

### 5.16 Cell Culture

The murine bladder cancer cell line MB49 (RRID: CVCL7076) was obtained from Millipore and was routinely tested for mycoplasma contamination. Cells were cultured in DMEM supplemented with 10% fetal bovine serum and 1% penicillin‐streptomycin at 37°C in a humidified incubator containing 5% CO_2_. Cells were passaged at 70%–80% confluence using 0.25% Trypsin‐EDTA (Thermo Scientific, Cat#25200072).

### 5.17 Transmission Electron Microscopy (TEM)

Treated macrophages were fixed with 2.5% glutaraldehyde in six‐well plates. After washing with phosphate buffer (0.1 m; pH 7.4), the samples were post‐fixed with 1% osmium tetroxide in phosphate buffer and washed three times with phosphate buffer (0.1 m; pH 7.4). Samples were then dehydrated, embedded, and incubated at 60°C for 48 h. Ultrathin sections were prepared and stained with uranyl acetate and lead citrate. After overnight drying, the sections were observed using an Olympus microscope.

### 5.18 Flow Cytometry (FC)

To assess the immune cell population in bladder cancer tissues, freshly isolated tumor tissues were digested in buffer containing collagenase II (Worthington‐biochem, Cat#LS004174) and DNase I (Worthington‐biochem, Cat#LS002139) to generate single‐cell suspensions, followed by antibody staining. For in vitro samples, cells were harvested and incubated with an AF700‐conjugated live/dead viability dye (BDBiosciences, Cat#564997) and antibodies against specific cell surface markers at 4°C for 30 min. For intracellular protein staining, cells were washed with PBS, fixed, permeabilized using permeabilization buffer (BioLegend, Cat#424401), and subsequently stained with the indicated antibodies. After staining, cells were washed three times with PBS, resuspended in PBS (200 µL), and analyzed on a Beckman flow cytometer. The antibodies used in this study were listed in Table S2.

### 5.19 GSH/GSSG Assay

Cellular GSH/GSSG levels were measured using a GSH/GSSG Assay Kit (Beyotime, Cat#S0053) according to the manufacturer's instructions. Briefly, cells were collected, washed with PBS, and lysed with protein removal reagent followed by rapid freeze–thaw cycles. After centrifugation at 4°C, the supernatants were collected for analysis. Total glutathione was measured with a freshly prepared total glutathione working solution. For GSSG measurement, samples were first treated with a GSH scavenging reagent and then incubated with the working solution. Total glutathione and GSSG concentrations were determined from standard curves at 412 nm. GSH content was calculated accordingly, and the GSH/GSSG ratio was used as an indicator of cellular redox status.

### 5.20 Cellular Thermal Shift Assay (CETSA)

Cells were harvested and washed twice with PBS containing a protease inhibitor cocktail (CWBio, Cat#CW2200s) and resuspended in the same buffer. Cell suspensions were aliquoted into fractions (100 µL) and heated at the indicated temperatures (37, 40, 43, 46, 49, 52, 55, 58°C) for 3 min. After heating, samples were subjected to three freeze–thaw cycles by snap‐freezing in liquid nitrogen followed by thawing on ice to achieve complete lysis. Soluble fractions were collected after centrifugation (13,000 × *g*; 30 min) and analyzed by immunoblotting.

### 5.21 Protein Extraction and Western Blotting

Cells were collected, washed three times with PBS, and lysed in RIPA buffer (EpiZyme, Cat#PC101) supplemented with protease and phosphatase inhibitor cocktails (CWBio, Cat#CW2383S) on ice for 30 min. After centrifugation (3,000 × *g*; 30 min), the supernatants were collected, and protein concentrations were measured using a BCA Protein Assay Kit (Thermo Scientific, Cat#23227). Equal amounts of protein were separated by 10% SDS–PAGE and transferred onto PVDF membranes. Membranes were blocked with 5% BSA and incubated overnight at 4°C with the indicated primary antibodies, followed by HRP‐conjugated secondary antibodies for 2 h at room temperature. Immunoreactive bands were detected using an ECL chemiluminescence kit (4A Biotech, Cat#4AW011).

### 5.22 siRNA Transfection

Cells were plated one day before transfection. Ahr siRNA or control siRNA was transfected using the CALNP RNAi in vitro transfection system (D‐Nano Therapeutics, Cat#DN001) following the manufacturer's protocol. Briefly, siRNA was sequentially combined with Reagent A and Reagent B to generate transfection complexes, which were then diluted in culture medium and added to cells. Cells were incubated under standard conditions (37°C, 5% CO_2_), and knockdown efficiency was evaluated at the protein level after 48 h. The sequences used in this study are listed in Table S3.

### 5.23 mRNA Transfection

Cells were seeded one day prior to transfection to reach appropriate confluency. Ahr mRNA was transfected using the CALNP mRNA in vitro transfection reagent (D‐Nano Therapeutics, Cat#DN002) according to the manufacturer's instructions. Briefly, mRNA was mixed sequentially with Reagent A and Reagent B to form mRNA–lipid complexes in complete culture medium, followed by incubation at room temperature. The transfection complexes were then added to BMDMs and gently mixed. BMDMs were cultured under standard conditions (37°C, 5% CO_2_), and gene expression was analyzed 24–48 h post‐transfection.

### 5.24 Preparation of Mannose‐Modified Liposomes

DSPC, cholesterol, and DSPE‐PEG2k‐Mannose were mixed (mass ratio of 8:1:1) and dissolved together with I3P and DiR in ethanol. The mixture was transferred to a pear‐shaped flask, and the organic solvent was removed by rotary evaporation to form a thin lipid film. Ultrapure water was then added, and the film was detached by bath sonication. The suspension was transferred to centrifuge tubes and further sonicated with a probe sonicator under cooling conditions to maintain the temperature below 20°C. The resulting liposomes were filtered through a 0.45 µm syringe filter, purified by ultrafiltration (900 rpm, 3 cycles), and then passed through a 0.22 µm syringe filter to remove aggregates. The final formulation was stored at 4°C until use.

### 5.25 Phagocytosis Assay of Macrophages

Tumor cells were labeled with CellTracker Green CMFDA (Thermo Scientific, Cat#C2925) according to the manufacturer's instructions. Macrophages were pretreated and co‐cultured with tumor cells (1:1) for 12 h. Cells were then stained with anti‐F4/80 (BioLegend, Cat#70076) and analyzed by flow cytometry, and phagocytosis was assessed as the proportion of CFSE^+^ cells within the F4/80^+^ population.

### 5.26 Cytotoxicity of CD8^+^ T Cells

Tumor cells were labeled with CellTracker Green CMFDA according to the manufacturer's instructions. CD8^+^ T cells were pretreated for 24 h with supernatants from the indicated macrophages and co‐cultured with tumor cells (effector‐to‐target ratio of 10:1) for 12 h. Cells were then stained with propidium iodide (BioLegend, Cat#640914) and analyzed by flow cytometry, with tumor cell death assessed in the CMFDA‐positive population.

### 5.27 Intracellular Fe^2+^ Measurement

A FerroOrange fluorescent probe (Servicebio, Cat# G1727) was used according to the manufacturer's instructions. BMDMs were pretreated with vehicle, free I3P (40 µg mL^−1^), or Nano@I3P (40 µg mL^−1^) for 24 h and washed with PBS. The cells were incubated with FerroOrange probe diluted in solution buffer (1:1000) at 37°C for 30 min. After washing with the solution buffer, the cells were analyzed by flow cytometry.

### 5.28 Cell Viability Assay

Cell viability was determined using a Cell Counting Kit‐8 kit (Biosharp, Cat#BS350D). Cells were seeded into 96‐well plates (5 × 10^3^ cells per well) and incubated overnight. After the indicated treatments, CCK‐8 solution was added to each well to a final concentration of 10%, and cells were further incubated for 2 h. Absorbance at 450 nm was measured using a microplate reader.

### 5.29 Colony Formation Assay

MB49 cells were seeded into 6‐well plates (5000 cells per well) and treated with vehicle or free I3P (200 µm). The culture medium was replaced every 3 days with fresh medium containing the indicated treatment. On day 14, adherent colonies were fixed with paraformaldehyde (4%) for 20 min and subsequently stained with crystal violet (0.1%) for 10 min. Colonies were then imaged with a light microscope.

### 5.30 Migration Assay

MB49 cells were resuspended in serum‐free DMEM medium and seeded into the upper chambers for migration, while DMEM medium with serum and vehicle or I3P (200 µm) was added to the lower chamber. After 24 h of incubation, non‐migrated cells were gently removed. Migrated cells on the lower surface were treated with paraformaldehyde (4%) and crystal violet (0.1%) for 20 min. Images were captured using a light microscope.

### 5.31 Wound Healing Assay

MB49 cells were seeded into 6‐well plates (5 × 10^5^ cells per well) with 2 mL DMEM. When cells reached approximately 90% confluence, a straight scratch was generated. The samples were gently washed twice with PBS and then treated with vehicle or free I3P (200 µm). The healing of the wounds was monitored at indicated time points, and images were captured using a light microscope.

### 5.32 Statistical Analysis

Statistical analyses were performed using GraphPad Prism 10.5. The results associated with *p* < 0.05 were considered statistically significant. Error bars indicate the S.D. of at least three independent experiments. An unpaired two‐tailed Student's t‐test was used for comparisons between two groups. Multiple *t*‐tests were used for comparisons between two groups at different stages. Nonparametric data were analyzed using the Mann–Whitney U test. Tumor growth curves were analyzed using two‐way repeated‐measures ANOVA. For multiple‐group comparisons, one‐way ANOVA followed by Tukey's multiple‐comparisons test was performed.

## Author Contributions

**Daqin Wu**: methodology, data curation. **Shuai Liang**: investigation, validation. **Kai Deng**: visualization, investigation. **Longhao Xu**: methodology, data curation. **Kun Zheng**: software. **Jianwen Lao**: investigation, writing – original draft, validation. **Peicong Cai**: formal analysis. **Junyu Chen**: supervision. **Xinhao Yuan**: investigation, validation. **Mingli Luo**: supervision. **Wenlong Zhong**: conceptualization, funding acquisition, resources, project administration, writing – review and editing. **Haoran Zheng**: formal analysis. **Chunhui Wang**: conceptualization, funding acquisition, writing – review and editing. **Yi Lin**: software. **Yan Chen**: supervision. **Xiong Chen**: funding acquisition, validation.

## Conflicts of Interest

The authors declare no conflict of interest.

## Supporting information




**Supporting File 1**: advs76319‐sup‐0001‐SuppMat.docx.

**Supporting File 2**: advs76319‐sup‐0002‐Tables S1‐S3.docx.

## Data Availability

The data that support the findings of this study are available from the corresponding author upon reasonable request.
