# Supplementary material for: Targeted Delivery of Indole‐3‐Pyruvic Acid Suppresses Macrophage Ferroptosis to Enhance CD8+ T Cell‐Mediated Immunotherapy Response in Bladder Cancer
Source: Adv Sci (Weinh). 2026 Jul 2:e76319. Online ahead of print. doi: 10.1002/advs.76319 (PMC13334596; doi:10.1002/advs.76319)
Supplement: Supplementary file 1 — Supporting File 1: advs76319‐sup‐0001‐SuppMat.docx. [file ADVS-9999-e76319-s001.docx]

**
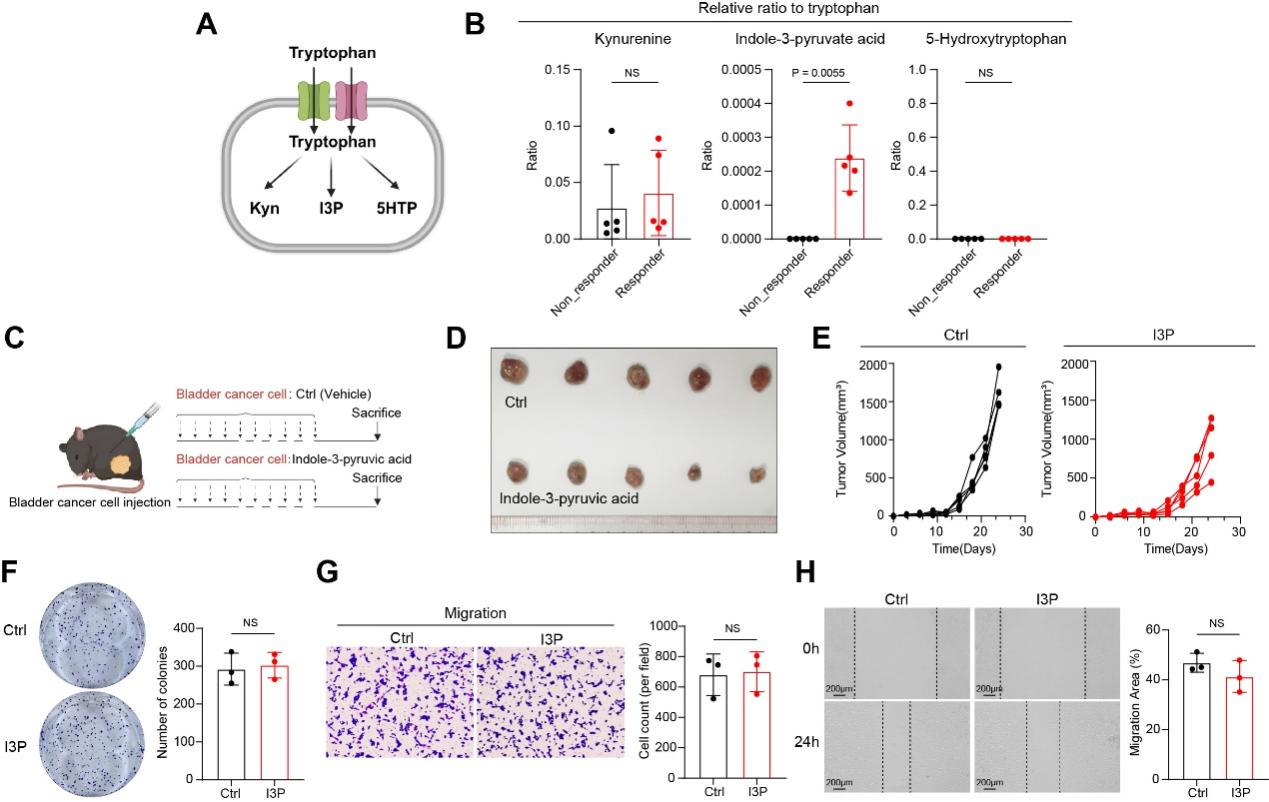
**

**Figure S1. Indole-3-pyruvic acid remodels the bladder cancer immune microenvironment.**

**(A)** Schematic overview of tryptophan metabolism, highlighting major catabolites including kynurenine (Kyn), indole-3-pyruvic acid (I3P), and 5-hydroxytryptophan (5-HTP).
**(B)** Relative ratios of tryptophan downstream metabolites to tryptophan in BCa from immunotherapy non-responders and responders.

**(C)** Experimental schematic of subcutaneous bladder cancer model treated with I3P (50 mg/kg, oral gavage, daily) or vehicle control.
**(D)** Representative gross images of tumors from the indicated groups.

**(E)** Tumor growth curves of indicated tumors in C57BL/6 mice treated with or without I3P.

**(F)** Colony formation assay of MB49 treated with 200 μM I3P or indicated vehicle
**(G)** Transwell migration assay of MB49 treated with 200 μM I3P or vehicle.
**(H)** Wound-healing assay of MB49 treated with 200 μM I3P or vehicle.


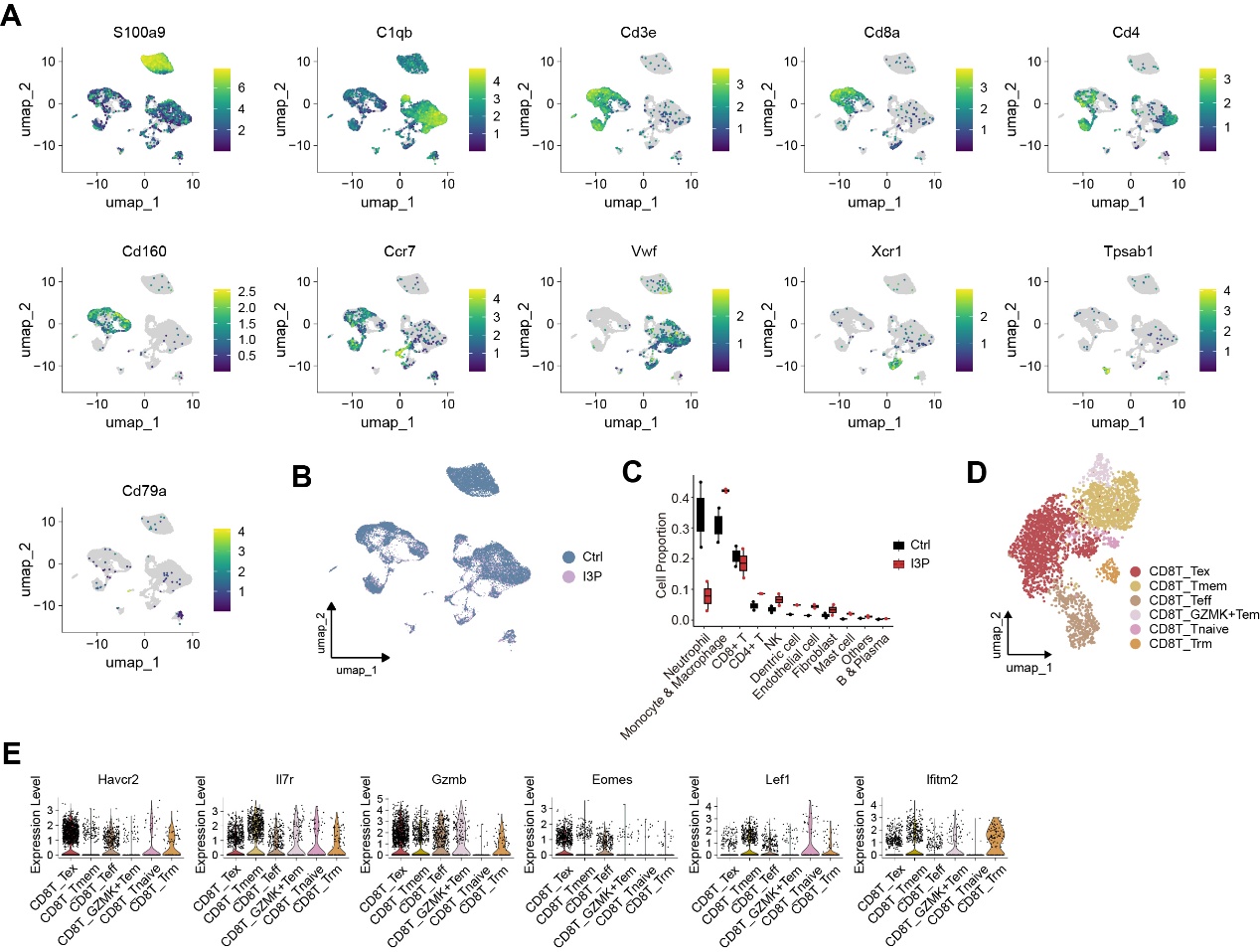


**Figure S2. Indole-3-pyruvic acid-induced single-cell transcriptomic landscape of tumor-infiltrating immune cells.**

**(A)** Density plots showing the expression of major cell-type markers across clusters identified by scRNA-seq.

**(B)** UMAP visualization of cells from different groups based on scRNA-seq analysis.

**(C)** Box plots showing the proportions of major cell types in different groups.

**(D)** UMAP visualization of CD8⁺ T cell subtypes identified by scRNA-seq.

**(E)** Violin plots showing the expression of major markers across CD8⁺ T cell clusters.

**
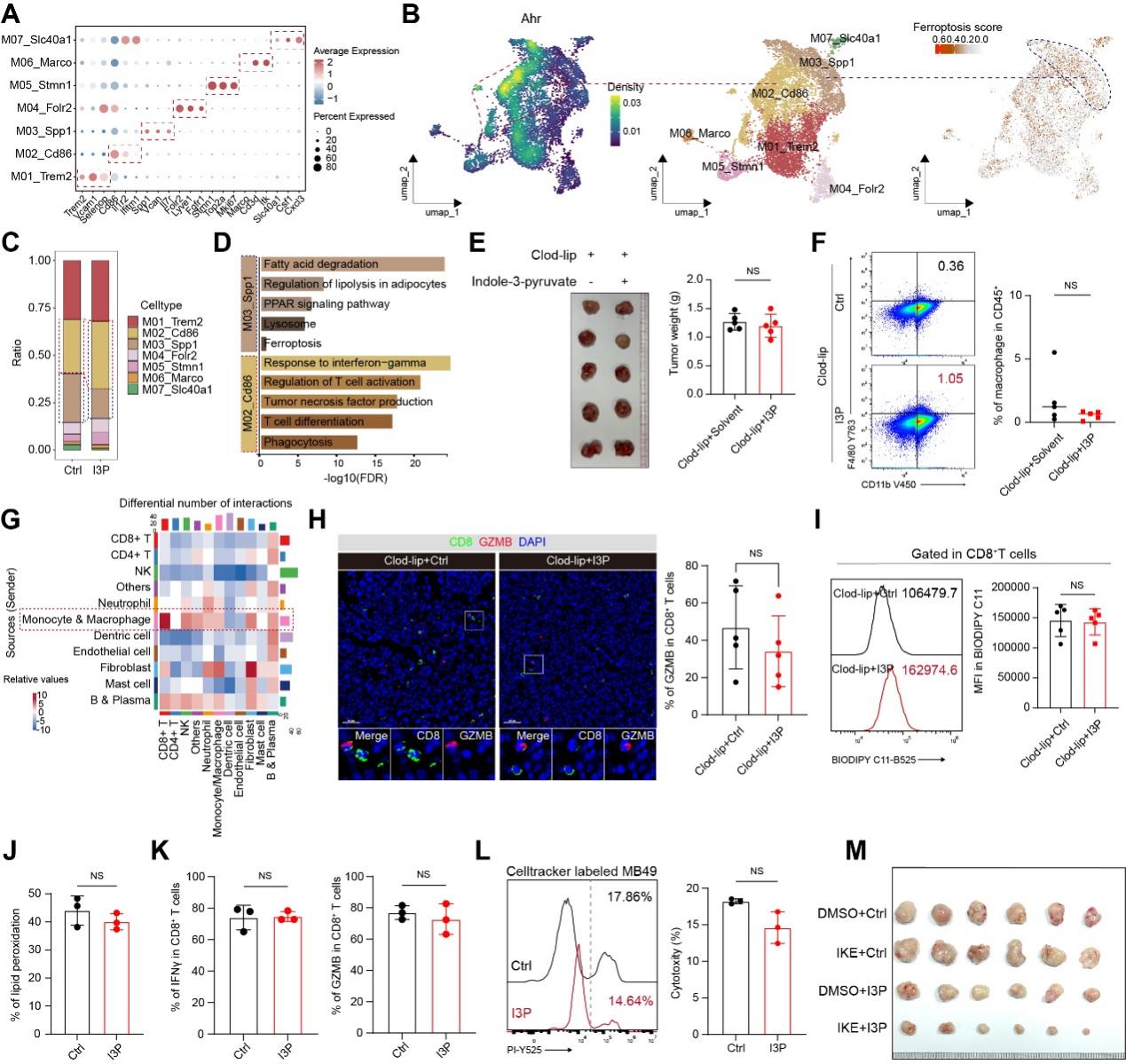
**

**Figure S3. Tumor-associated macrophages are the primary target cells of Indole-3-pyruvic acid.**

**(A)** Dotplot showing the expression of cell markers across macrophages subclusters.

**(B)** UMAP plot and density plot showing the association between Ahr expression and ferroptosis score across macrophages.

**(C)** Bar plot showing macrophages re-clustering based on scRNA-seq analysis in tumors treated with or without I3P.

**(D)** Enriched pathways based on different genes in the M03_Spp1 and M02_Cd86 macrophages subsets.

**(E)** Representative gross images and tumor weight from the indicated groups with macrophages depletion.

**(F)** Flow cytometric analysis of the F4/80⁺ macrophages infiltration following the indicated treatments.

**(G)** CellChat analysis of intercellular communication among major cell types.

**(H)** Representative mIF images and quantification of GZMB in CD8^+^ T cells.

**(I)** Flow cytometric analysis of lipid peroxidation in tumor-infiltrating CD8^+^ T cells following the indicated treatments.

**(J-K)** Flow cytometric analysis of lipid peroxidation **(J)** and cytotoxicity **(K)** in CD8⁺ T cells following 200μM I3P or vehicle treatments in vitro.

**(L)** Representative flow cytometry histogram and quantification of tumor cell killing by CD8^+^ T cells with direct 200μM I3P treatment.

**(M)** Representative gross images of subcutaneous BCa treated with or without IKE and I3P.

**
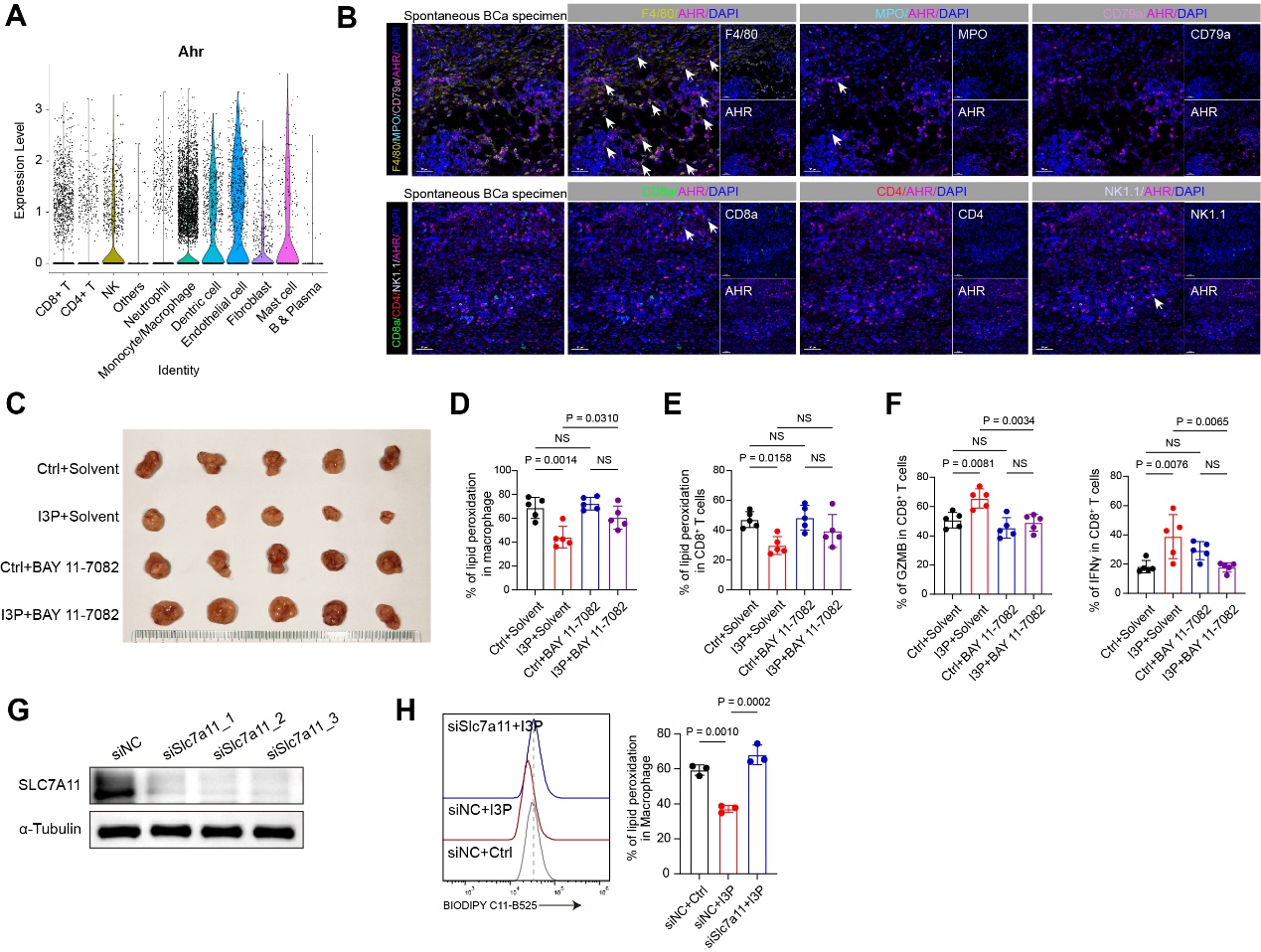
**

**Figure S4. AHR–NF-κB-SLC7A11 signaling is required for I3P-mediated effects in macrophages.**

**(A)** Violin plot showing Ahr expression across different cell types in subcutaneous BCa.

**(B)** Representative mIF images showing AHR expression in major immune cell populations in spontaneous BCa.

**(C)** Representative gross images of subcutaneous BCa treated with or without I3P and BAY 11-7082.

**(D)** Flow cytometric analysis of lipid peroxidation in macrophages following the indicated treatments.

**(E)** Flow cytometric analysis of lipid peroxidation in CD8^+^ T cell following the indicated treatments.

**(F)** Flow cytometric analysis of GZMB and IFN-γ in CD8^+^ T cell following the indicated treatments.

**(G)** Western blotting validation of Slc7a11 knockdown efficiency in si Slc7a11-transfected BMDMs.

**(H)** Representative flow cytometry histogram and quantification of lipid peroxidation in BMDMs treated with I3P after siSlc7a11 transfection.

**
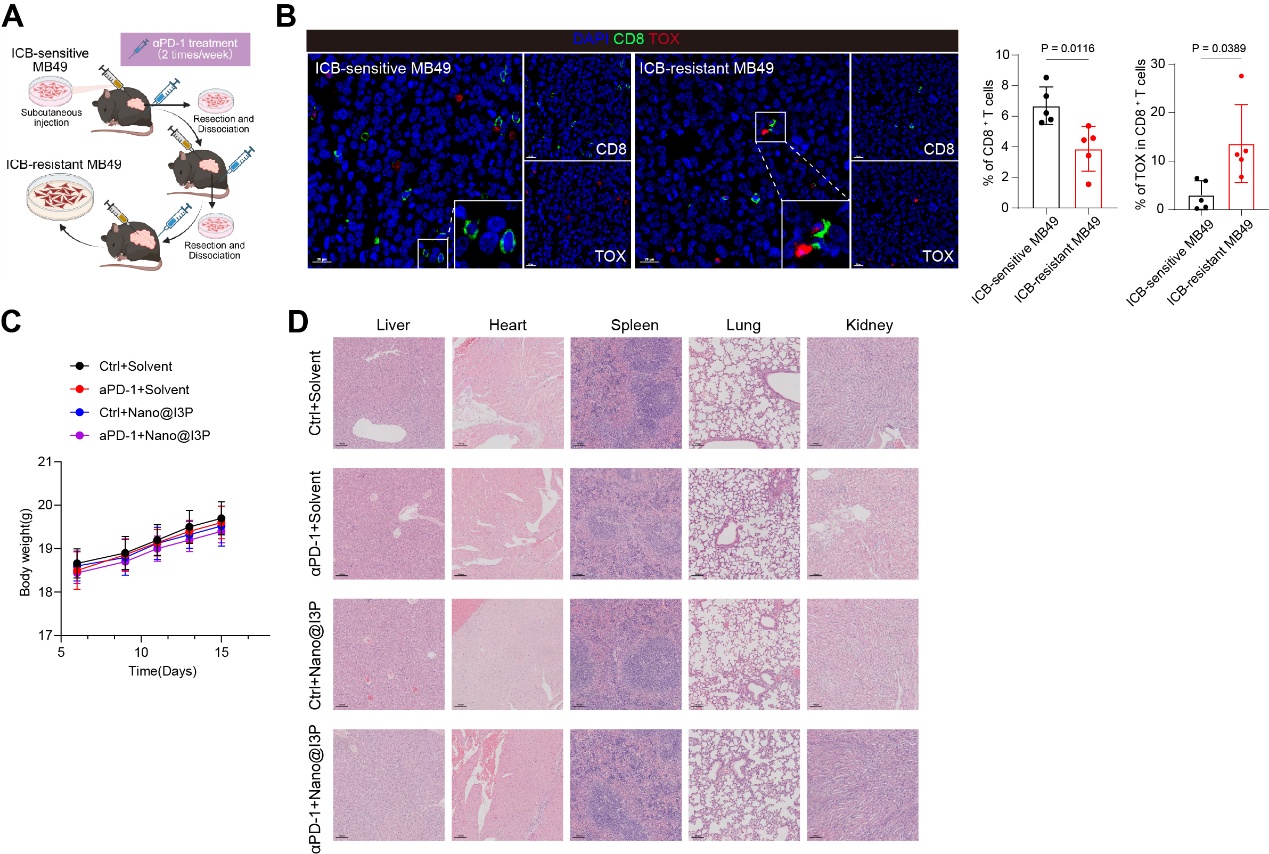
**

**Figure S5. Characterization of the ICB-resistant BCa model and Nano@I3P biosafety.**

**(A)** Schematic of the ICB-resistant MB49 subcutaneous BCa model generated through three rounds of in vivo αPD-1 treatment.

**(B)** Representative mIF images showing increased TOX expression and impaired CD8⁺ T cell infiltration in ICB-resistant MB49-derived BCa, indicative of an immune-cold phenotype.

**(C)** Body weight curves of mice in different treatment groups over the course of the study.

**(D)** Representative H&E staining of the liver, heart, spleen, lung, and kidney from mice treated with Ctrl+Solvent, αPD-1+Solvent, Ctrl+Nano@I3P, or αPD-1+Nano@I3P.
