# Supplementary material for: Targeted Delivery of Indole‐3‐Pyruvic Acid Suppresses Macrophage Ferroptosis to Enhance CD8+ T Cell‐Mediated Immunotherapy Response in Bladder Cancer
Source: Adv Sci (Weinh). 2026 Jul 2:e76319. Online ahead of print. doi: 10.1002/advs.76319 (PMC13334596; doi:10.1002/advs.76319)
Supplement: Supplementary file 2 — Supporting File 2: advs76319‐sup‐0002‐Tables S1‐S3.docx. [file ADVS-9999-e76319-s002.docx]

**Supplementary Table 1. Baseline clinicopathological characteristics** **for patients in SYSMH cohorts. ^a^**

N-, negative lymph nodes metastasis; N+, positive lymph nodes metastasis. P values reported are two-tailed from Pearson’s chi-square test or Fisher’s exact test.

**^a^** Data are presented as number (percentage) of patients unless otherwise indicated.

| **Characteristics** | **Non_responder**  **(n=17)** | **Responder**  **(n=17)** | **P value** |
| --- | --- | --- | --- |
| **Age, mean (SD), years** | 62.8 (10.6) | 63.0 (11.7) | 0.959 |
| **Gender** |  |  | 1.000 |
| Male | 14 (82.4%) | 14 (82.4%) |  |
| Female | 3 (17.6%) | 3 (17.6%) |  |
| **Pathologic T stage** |  |  | 1.000 |
| <T2 | 14 (82.4%) | 15 (88.2%) |  |
| ≥T2 | 3(17.6%) | 2 (11.8%) |  |
| **Pathologic N status** |  |  | 1.000 |
| N- | 13 (76.5%) | 12 (70.6%) |  |
| N+ | 4 (23.5%) | 5 (29.4%) |  |
| **Pathologic grade** |  |  | 1.000 |
| Low | 0 (0.0%) | 1 (5.9%) |  |
| High | 17 (100.0%) | 16 (94.1%) |  |
| **Tumor size** |  |  | 1.000 |
| ≤ 3 cm | 12 (70.6%) | 13 (76.5%) |  |
| > 3 cm | 5 (29.4%) | 4 (23.5%) |  |
| **Tumor multifocality** |  |  | 1.000 |
| Unifocal | 7 (41.2%) | 8 (47.1%) |  |
| Multifocal | 10 (58.8%) | 9 (52.9%) |  |

**Supplementary Table 2. Antibodies used in this study.**

| **Antibodies** | **Source** | **Identifier** |
| --- | --- | --- |
| InVivoMAb anti-mouse PD-1 | Selleck | Cat#A2122;  RRID: AB_3644244 |
| Rabbit anti-human CD68 | Cell Signaling Technology | Cat#76437;  RRID: AB_2799882 |
| Rabbit anti-mouse CD8α | Cell Signaling Technology | Cat#98941;  RRID: AB_2756376 |
| Rabbit anti-mouse F4/80 | Cell Signaling Technology | Cat#70076;  RRID: AB_2799771 |
| Rabbit anti-mouse CD45 | Cell Signaling Technology | Cat#70257;  RRID: AB_2799780 |
| Goat anti-mouse Myeloperoxidase | R&D Systems | Cat#AF3667;  RRID: AB_2250866 |
| Rabbit anti-mouse CD79a | Proteintech | Cat#22349-1-AP;  RRID: AB_2879084 |
| Rabbit anti-mouse CD4 | Cell Signaling Technology | Cat#25229;  RRID: AB_2798898 |
| Rabbit anti-mouse NK1.1 | Cell Signaling Technology | Cat#39197;  RRID: AB_2892989 |
| Mouse anti-mouse Pan-Keratin | Abcam | Cat#ab8068;  RRID: AB_306238 |
| Rabbit anti-mouse GZMB | Cell Signaling Technology | Cat#46890;  RRID: AB_2799313 |
| Mouse anti 4-HNE | Abcam | Cat#ab48506;  RRID: AB_867452 |
| Rabbit anti-mouse AHR | Abcam | Cat#ab308215;  RRID: AB_3083080 |
| Rabbit anti-mouse SLC7A11 | Cell Signaling Technology | Cat#98051;  RRID: AB_2800296 |
| Rabbit anti-mouse Phospho-IKK alpha/beta (Ser176/180) | Cell Signaling Technology | Cat#2697;  RRID: AB_2079382 |
| Rabbit anti-mouse Phospho-NF-kappaB (Ser536) | Cell Signaling Technology | Cat#3033;  RRID: AB_331284 |
| Rabbit anti-mouse IKK alpha | Cell Signaling Technology | Cat#2682;  RRID: AB_331626 |
| Rabbit anti-mouse IkappaB alpha | Cell Signaling Technology | Cat#4812;  RRID: AB_10694416 |
| Rabbit anti-mouse NF-kappaB | Cell Signaling Technology | Cat#8242;  RRID: AB_10859369 |
| Rabbit anti-mouse alpha-Tubulin | Cell Signaling Technology | Cat#2144;  RRID: AB_2210548 |
| Rabbit anti-mouse GAPDH | Cell Signaling Technology | Cat#2118;  RRID: AB_561053 |
| Fixable Viability Stain 700 | BDBiosciences | Cat#564997;  RRID: AB_2869637 |
| Rat anti-mouse CD8a-APC-Cy7 | Biolegend | Cat#100713;  RRID: AB_312752 |
| Mouse anti-mouse CD45-PE | Biolegend | Cat#157603;  RRID: AB_2876536 |
| Rat anti-mouse CD3-BV605 | Biolegend | Cat#100237;  RRID: AB_2562039 |
| Rat anti-mouse CD4-BV650 | Biolegend | Cat#100545;  RRID: AB_11126142 |
| Rat anti-mouse CD11b-BV421 | Biolegend | Cat#101235;  RRID: AB_10897942 |
| Rat anti-mouse F4/80-BV510 | Biolegend | Cat#123135;  RRID: AB_2562622 |
| Rat anti-mouse Ly6G-PC5.5 | Biolegend | Cat#127615;  RRID: AB_1877272 |
| Mouse anti-mouse NK1.1-APC | Biolegend | Cat#156505;  RRID: AB_2876525 |
| Mouse anti-mouse GZMB-FITC | Biolegend | Cat#372205;  RRID: AB_2687029 |
| Rat anti-mouse IFNγ-PE | Biolegend | Cat#505807;  RRID: AB_315401 |

**Supplementary Table 3. Sequences used in this study.**

| **Identifier** | **Source** | **Sequence** |
| --- | --- | --- |
| siAhr#1 | OBio Technology | Sense: GGUCCGAAGCACACGCAAATT  Antisense: UUUGCGUGUGCUUCGGACCTT |
| siAhr#2 | OBio Technology | Sense: CCUCCACUAUCCAAGAUUATT  Antisense: UAAUCUUGGAUAGUGGAGGTT |
| siAhr#3 | OBio Technology | Sense: GAGCAUUUACAGAAGCGAATT  Antisense: UUCGCUUCUGUAAAUGCUCTT |
| siNC | OBio Technology | Sense: UUCUCCGAACGUGUCACGUTT  Antisense: ACGUGACACGUUCGGAGAATT |
| siSlc7a11#1 | OBio Technology | Sense: GAGUUAUACAGCUAAUUAATT  Antisense: UUAAUUAGCUGUAUAACUCTT |
| siSlc7a11#2 | OBio Technology | Sense: GAUUUAUCUUCGAUACAAATT  Antisense: UUUGUAUCGAAGAUAAAUCTT |
| siSlc7a11#3 | OBio Technology | Sense: GAAUUAGGUACAAGCAUAATT  Antisense: UUAUGCUUGUACCUAAUUCTT |
